# Biopolymer-Based Hydrogels for Wound Healing: Advances in Cellulose, Chitosan, Alginate, and Hyaluronic Acid from Design to Clinical Translation

**DOI:** 10.3390/ph19081210

**Published:** 2026-08-01

**Authors:** Shery Jacob, Namitha Raichel Varkey, Sai H. S. Boddu, Jigar N. Shah, Rekha Rao, Anroop B. Nair

**Affiliations:** 1 Department of Pharmaceutical Sciences, College of Pharmacy, Gulf Medical University, Ajman 4184, United Arab Emirates; 2025mdd01@mygmu.ac.ae; 2 Department of Pharmaceutical Sciences, College of Pharmacy and Health Sciences, Ajman University, Ajman 346, United Arab Emirates; s.boddu@ajman.ac.ae; 3 Center of Medical and Bio-Allied Health Sciences Research, Ajman University, Ajman 346, United Arab Emirates; 4 Department of Pharmaceutics, Institute of Pharmacy, Nirma University, Ahmedabad 382481, India; jigar.shah@nirmauni.ac.in; 5 Department of Pharmaceutical Sciences, Guru Jambheshwar University of Science and Technology, Hisar 125001, India; rekhaline@gjust.org; 6 Department of Pharmaceutical Sciences, College of Clinical Pharmacy, King Faisal University, Al-Ahsa 31982, Saudi Arabia; anair@kfu.edu.sa

**Keywords:** wound healing, biopolymer hydrogels, carboxymethyl cellulose, hydroxyethyl cellulose, hydroxypropyl methylcellulose, chitosan, alginate, hyaluronic acid, patents, clinical trials

## Abstract

Wound healing is a multifaceted biological process comprising the phases of hemostasis, inflammation, proliferation, and remodeling, all of which require supportive microenvironment for optimal tissue regeneration. Biopolymer-based hydrogels, derived from materials such as cellulose and its derivatives, chitosan, alginate, and hyaluronic acid, have emerged as promising wound dressing materials due to their excellent biocompatibility, biodegradability, moisture-retention capacity, and potential to mimic the native extracellular matrix. The structural characteristics, wound healing functions, and underlying mechanisms of these biopolymers are critically examined and summarized in tabular form. The review further highlights the incorporation of natural and synthetic therapeutic agents, growth factors, stem-cell-derived products, and peptides into biopolymer matrices to enhance therapeutic efficacy. The examined research findings indicate significant increases in fluid intake, moisture retention, antibacterial activity, angiogenesis, collagen deposition, tissue regeneration, and wound healing rates. Translational difficulties, regulatory issues, clinical research, and new patent activity pertaining to advanced wound healing biomaterials are also covered in the review. Despite tremendous improvements, issues still exist in bulk manufacturing, long-term safety, reproducibility, mechanical stability, and clinical validation. Future innovations are anticipated to concentrate on smart, multipurpose, and customized hydrogel systems that can integrate drug delivery, biosensing, and regenerative capabilities while reacting dynamically to wound microenvironments. Overall, biopolymer-based hydrogels are a flexible, rapidly developing platform with significant promise to improve next-generation skin tissue engineering and change the treatment of both acute and chronic wounds.

## 1. Introduction

Hydrogels are now regarded as one of the most appropriate wound dressing materials, especially for burn therapy, owing to their ability to precisely replicate the physiological environment of skin tissue. They can be readily tailored to conform to irregular tissue architectures, thereby facilitating tissue regeneration, angiogenesis, and extracellular matrix (ECM) remodeling, and have demonstrated promising outcomes in wound healing applications [1]. Their water content enables maintenance of optimal moist environment, which is critical for accelerating epithelial cell migration and proliferation. It is important to note that while moist environment is essential for optimal wound healing, excessive moisture can hinder the healing process and delay recovery [2]. For instance, over-accumulation of wound exudate may lead to periwound maceration, degradation of newly formed tissue, delayed epithelialization, increased bacterial proliferation, and frequent dressing replacement, particularly in highly exuding chronic wounds [3,4]. Therefore, the primary objective of hydrogel dressings is not to maximize hydration but to maintain an optimal moisture balance by retaining sufficient water at the wound interface while efficiently managing excess exudate [5]. Modern hydrogel dressings achieve this balance through controlled swelling behavior, optimized crosslinking density, and appropriate oxygen and water vapor transmission rates (WVTR), enabling effective absorption of wound fluid without compromising structural integrity or causing wound dehydration. Furthermore, advanced hydrogel systems incorporating superabsorbent polymers, multilayer structures, or stimuli-responsive networks can dynamically regulate fluid uptake according to exudate levels, thereby minimizing the risk of wound maceration while maintaining a favorable microenvironment for tissue regeneration [6]. Consequently, optimizing swelling capacity, mechanical stability, and exudate-handling properties represents a critical design consideration for the successful clinical translation of hydrogel wound dressings, particularly for diabetic foot ulcers, venous leg ulcers, and pressure injuries. Hydrogels possess inherent biocompatibility, a soft and elastic nature, and structural similarity to the ECM, which enhance patient comfort and minimize mechanical irritation during application and removal. Furthermore, hydrogels provide cooling effect that alleviates pain, and minimize disturbance to newly formed tissue, thereby supporting uninterrupted healing processes [7,8]. From materials science perspective, the traditionally poor mechanical strength of hydrogels has been significantly improved through the development of composite and hybrid hydrogel systems. Hydrogels may quickly lose water in real-life situations, which would limit their capacity to keep moist environment at the wound site [9]. The hydrogel may become dry and rigid as a result of this dehydration, which could result in weak adhesion and ultimately separate from the surface of the skin. In order to minimize moisture loss in clinical settings, hydrogel dressings are frequently combined with hydrophobic film as secondary layer [10]. However, the low air permeability of these hydrophobic membranes may prevent effective wound healing. In addition to using sophisticated crosslinking techniques like physical, chemical, and irradiation methods, hydrogel systems frequently include reinforcing agents like nanoparticles, biopolymers (chitosan and alginate), or synthetic polymers such as polyvinyl alcohol (PVA), polyethylene glycol (PEG), and polyurethane (PU). These changes ensure high swelling capacity while enhancing structural integrity. The resulting three-dimensional polymeric networks are more suitable for clinical applications due to their better durability, variable porosity, and high water retention [11]. Despite their low antigenicity, biocompatible polymers can still induce immunological reactions. They may still cause foreign body reaction even after their surface and biochemical properties have been altered to reduce this effect [12].

Traditional wound dressings like gauze, cotton wool, and bandages are preferred due to their low cost and convenience of use. However, there are substantial disadvantages that limit their efficacy, especially in chronic and severe wounds. Traditional dressings frequently struggle to sustain proper moist wound environment, provide inadequate moisture balance, and lack inherent bioactive qualities such as antibacterial, anti-inflammatory, and regenerative capabilities [13,14]. Furthermore, dressing adherence to the wound surface might result in discomfort, hemorrhage, and subsequent tissue injury during removal [15]. Conventional dressings cannot replicate the ECM or provide controlled distribution of therapeutic drugs, which limits their potential to assist cellular proliferation, angiogenesis, and tissue regeneration [16]. As a result, the demand for better wound care materials has led to the development of hydrogel-based dressings that provide higher moisture retention, biocompatibility, bioactivity, and improved wound healing efficacy [14,17]. The current review includes recent breakthroughs in hydrogel-based systems for skin tissue engineering and wound healing, emphasizing their therapeutic potential.

Although several excellent review articles have summarized hydrogel dressings or individual biopolymers for wound healing, most have focused on specific biomaterials, hydrogel fabrication strategies, bioactive agents, or general wound healing applications [16,18,19,20,21,22]. Comparative analyses integrating the structural characteristics, biological functions, therapeutic mechanisms, and clinical relevance of the major natural biopolymers used in wound healing remain limited. Furthermore, previous reviews have devoted relatively little attention to recent advances in multifunctional and stimuli-responsive hydrogels, the incorporation of stem cell-derived products, peptides, growth factors, and natural and synthetic therapeutics, as well as emerging clinical evidence, the patent landscape, regulatory considerations, and translational challenges that govern successful commercialization [5,23,24]. To highlight the novelty and scope of the present review, Table 1 compares representative review articles on biopolymer hydrogels for wound healing published over the last five years. Unlike previous reviews focusing on individual biopolymers or selected aspects of hydrogel design, the present review provides a comprehensive and up-to-date overview of cellulose and its derivatives, chitosan and its derivatives, alginate, and hyaluronic acid-based hydrogels, highlighting their structure–function relationships, molecular mechanisms of wound repair, recent formulation advances, therapeutic functionalization strategies, and comparative advantages and limitations. In addition, it critically discusses safety considerations, clinical trials, intellectual property trends, manufacturing challenges, clinical translation, biopolymer-based bioinks for 3D bioprinting, carrier-free therapeutic hydrogels, and future opportunities for developing next-generation multifunctional wound dressings, thereby providing an integrated perspective for researchers, clinicians, and pharmaceutical scientists.

The literature included in this review was identified through comprehensive searches of major scientific databases, including PubMed, Scopus, Web of Science, Google Scholar, and ScienceDirect. Publications available up to June 2026 were considered. The search strategy employed combinations of keywords such as “biopolymer hydrogel”, “wound healing”, “hydrogel wound dressing”, “cellulose hydrogel”, “chitosan hydrogel”, “alginate hydrogel”, “hyaluronic acid hydrogel”, “stimuli-responsive hydrogel”, “drug delivery”, and “tissue regeneration”. Original research articles, review articles, clinical studies, and relevant patents published in English were screened for relevance. Studies focusing on the design, physicochemical properties, biological performance, therapeutic applications, clinical translation, and recent advances in biopolymer-based hydrogel wound dressings were included, whereas studies unrelated to wound healing, duplicate publications, conference abstracts without full text, and articles with insufficient experimental information were excluded.

## 2. Wound Healing Biology

### 2.1. Human Skin: Structure and Function

The epidermis, dermis, and hypodermis make up the complex, multilayered skin, which is made up of highly ordered tissue that is rich in ECM. Keratinocytes, which make up the majority of the epidermis, form an immunological and physical barrier that defends against pathogen invasion and excessive water loss. The dermis consists of connective tissue, fibroblasts, blood vessels, nerves, and ECM elements like collagen and elastin, which provide structural support, mechanical strength, and elasticity [17]. The hypodermis, which is mainly made up of adipose tissue and loose connective tissue, has multiple functions, including insulation, cushioning, energy storage, and metabolic regulation. Apart from its barrier function, the skin plays important roles in thermoregulation, sensory perception, immunological defense, vitamin D production, and wound healing [30].

Acute wounds usually follow structured healing process, undergoing phases of hemostasis, inflammation, proliferation, and remodeling over definite period of time. They typically occur due to trauma, surgery, or burns [13]. On the other hand, underlying pathological problems like infection, poor vascularization, and metabolic disorders cause chronic wounds like diabetic ulcers, pressure ulcers, and venous leg ulcers that fail to heal through the typical stages [30]. Conventional treatment for significant skin loss and chronic non-healing wounds typically includes split and full thickness skin grafts, along with skin flaps, tissue expansion procedures, and dermal replacements [17]. These methods are restricted by number of disadvantages despite their widespread clinical use and efficacy. This includes the scarcity of donor sites, their susceptibility to infection, the possibility of graft failure, and the risk of hypertrophic scarring or keloid formation, which could cause both functional constraints and psychological stress [15].

### 2.2. Stages of Wound Healing

**Figure 1:**
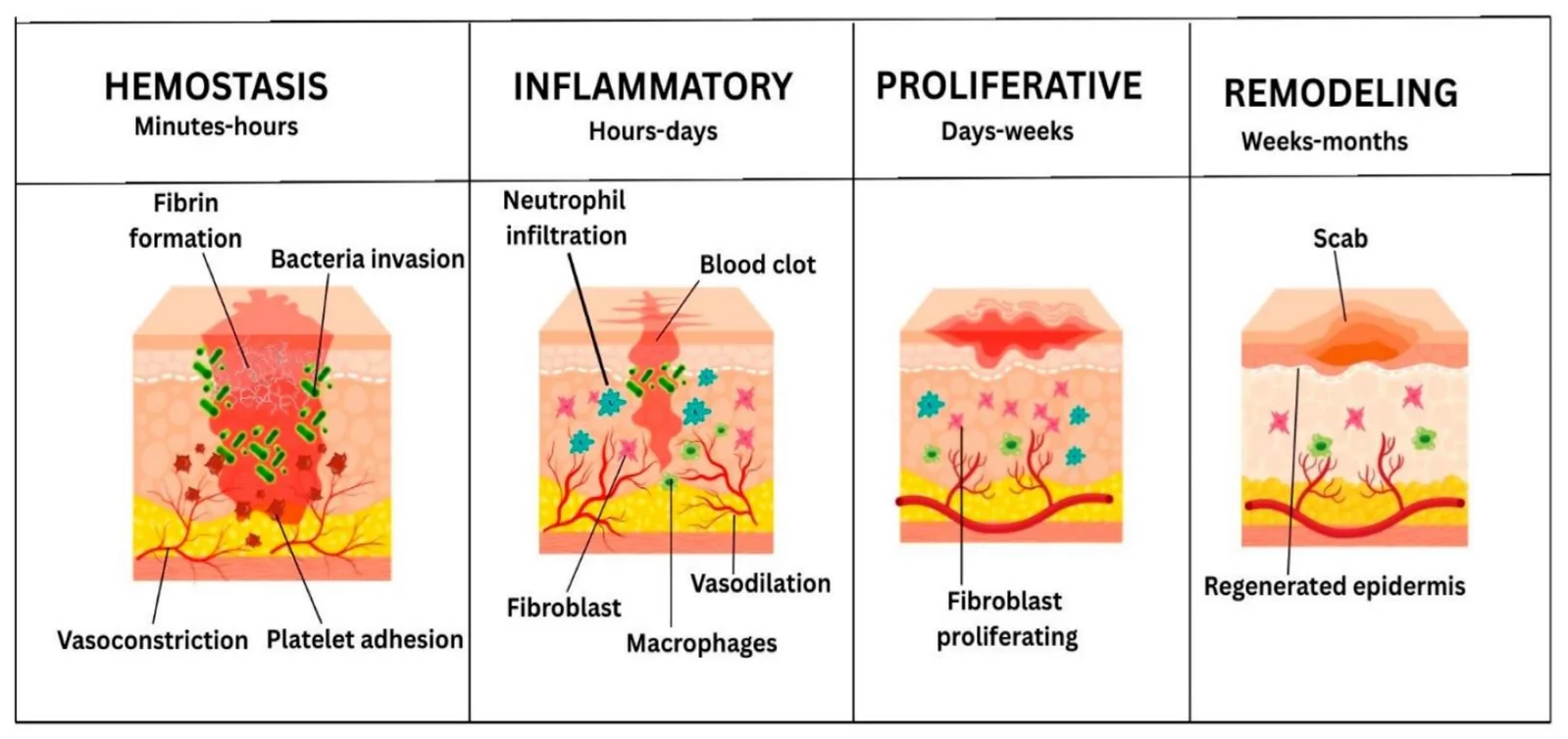
Schematic representation of the sequential phases of wound healing. Reproduced from Ref. [34].

Wound healing is a highly coordinated and dynamic biological process that progresses through sequential yet overlapping phases, including hemostasis, inflammation, proliferation, and maturation/remodeling (Figure 1). The hemostasis phase involves vasoconstriction, platelet aggregation leading to the formation of temporary platelet plug [31]. Platelets further facilitate the coagulation cascade, resulting in thrombin generation and the subsequent conversion of fibrinogen into crosslinked fibrin, which stabilizes the platelet plug. In this stage, platelets release growth factors such as platelet-derived growth factor (PDGF), transforming growth factor-beta (TGF-β), and vascular endothelial growth factor (VEGF). This is followed by inflammation, characterized by cellular recruitment and activation. This phase is marked by vasodilation, enhanced vascular permeability, and the release of inflammatory mediators such as histamine, prostaglandins, inflammatory cytokines, including interleukin-1 (IL-1) and interleukin-6 (IL-6), tumor necrosis factor-alpha (TNF-α), and chemokines that facilitate leukocyte recruitment and activation of the immune response. The proliferative phase involves granulation tissue formation, angiogenesis mediated by TGF-β, fibroblast growth factor (FGF), epidermal growth factor (EGF), and VEGF, re-epithelialization, and immune modulation [32]. The final maturation and remodeling phase, regulated by TGF-β, matrix metalloproteinases (MMP), and tissue inhibitors of metalloproteinases, leads to reorganization of the ECM, restoration of tissue integrity, and ultimately wound closure [33]. Each phase is tightly regulated by cytokines, growth factors, and cellular interactions, and disruption at any stage can impair healing outcomes.

### 2.3. Skin Substitutes

Skin substitutes constitute diverse and heterogeneous class of wound dressing systems that can be applied to the wound site to restore barrier function, promote tissue regeneration, and facilitate wound healing [35]. Depending on their composition and design, these substitutes may provide temporary coverage to protect the wound and support healing or serve as permanent replacements by integrating with host tissue and promoting neovascularization and ECM formation. Skin substitutes are broadly classified into three categories: Class I (temporary, acellular dressings) that provide protection and moisture retention, Class II (single-layer substitutes) designed to replace either the epidermis or dermis, and Class III (composite substitutes) that incorporate both layers and include autografts, allografts, xenografts, and tissue-engineered skin constructs [36]. Moreover, advanced skin substitutes may incorporate features such as controlled drug release, antimicrobial activity, and stimuli responsiveness to enhance therapeutic outcomes. Skin tissue engineering is rapidly advancing interdisciplinary field aimed at developing functional skin substitutes for clinical applications, particularly in the management of extensive burns, chronic wounds, and skin defects [37].

Modern 3D bioprinting facilitates the exact spatial placement of biomaterials and living cells, making it feasible to produce skin grafts with structures and functions that nearly resemble native tissue [38]. Recent advances in 3D bioprinting have highlighted the complementary roles of natural and synthetic polymeric hydrogels as bioinks. Natural polymers provide excellent biocompatibility and bioactivity, whereas synthetic polymers offer superior mechanical strength and printability. Their combination enables the fabrication of biomimetic constructs with enhanced structural integrity and regenerative potential for wound healing and skin tissue engineering [39]. A variety of natural and synthetic biomaterials have been investigated for the fabrication of skin substitutes [40]. Among them, hydrogel-based systems received a lot of interest in wound healing applications owing to their high water content, biocompatibility, and capacity to closely resemble the native ECM, which facilitates tissue regeneration, cellular infiltration, and nutrient exchange [16]. Hydrogels are ideal for advanced wound care due to the fact that they produce moist environment, promote gas permeability, and assist in critical healing processes like angiogenesis and cell proliferation. Additionally, their structural adaptability makes it possible to produce injectable, sprayable, or in situ forming dressings, which enhances adherence to uneven wound geometries and allows for less-invasive application. The development of smart hydrogel systems and nanocomposites, which are produced by adding nanoparticles or bioactive compounds to the hydrogel matrix, has been the focus of recent research.

These hybrid formulations overcome the drawbacks of traditional hydrogels and expedite wound healing by exhibiting increased mechanical strength, antibacterial activity, anti-inflammatory properties, and stimuli-responsive behavior [41]. Furthermore, hydrogel-nanoparticle platforms permit dynamic interaction with the wound environment and control drug delivery, which is a promising approach for next-generation, multipurpose wound dressings [42].

### 2.4. Pathological Characteristics of Chronic Wounds

Chronic wounds are characterized by a dysregulated healing process in which tissue repair becomes arrested in the inflammatory phase, resulting in delayed or incomplete healing [43]. Their microenvironment is marked by persistent infiltration of neutrophils and pro-inflammatory macrophages, excessive production of inflammatory cytokines (e.g., TNF-α, IL-1β, and IL-6), elevated MMP activity, reduced bioavailability of growth factors, impaired angiogenesis, cellular senescence, and bacterial biofilm formation, all of which collectively hinder tissue regeneration [44]. Furthermore, chronic wounds exhibit sustained oxidative stress caused by excessive accumulation of reactive oxygen species (ROS). Although physiological levels of ROS are essential for antimicrobial defense and cell signaling, prolonged ROS overproduction damages cellular lipids, proteins, and DNA, disrupts ECM remodeling, and perpetuates inflammation, thereby maintaining a non-healing wound microenvironment [45,46]. These pathological features have driven the development of advanced hydrogel dressings that not only maintain an optimal moist wound environment but also actively modulate the wound microenvironment through antioxidant, anti-inflammatory, antimicrobial, immunomodulatory, and pro-angiogenic functions. Accordingly, contemporary hydrogel systems increasingly incorporate ROS-scavenging nanomaterials, bioactive polymers, growth factors, cytokine-regulating agents, and stimuli-responsive therapeutic platforms to overcome the biological barriers associated with chronic wound healing and promote functional tissue regeneration [5,16].

## 3. Hydrogels for Wound Healing

### 3.1. Classification of Hydrogels

Hydrogels are three-dimensional networks of hydrophilic polymers that can absorb and retain large quantities of water or biological fluids without compromising their structural integrity. Based on their origin, crosslinking process, and sensitivity to external stimuli, hydrogels can be classified. They are basically three categories based on their origin, synthetic hydrogels (PVA and PEG), natural hydrogels (cellulose, chitosan, alginate, hyaluronic acid (HA), collagen, and gelatin), and hybrid hydrogels that contain synthetic and natural polymers [14]. Depending on the kind of crosslinking, hydrogels can be chemically crosslinked through covalent bonds that offer more mechanical stability, or physically crosslinked by hydrogen bonds, ionic contacts, or hydrophobic associations. It was reported that soft, lightly crosslinked hydrogels promote greater cellular infiltration, reduced inflammation, improved tissue integration, and smaller scar formation, whereas heavily crosslinked hydrogels induce stronger inflammatory and pro-fibrotic responses [47]. Furthermore, advanced hydrogels can be classified as stimuli-responsive or smart hydrogels, capable of responding to pH, temperature, enzymes, light, or other environmental triggers to achieve controlled therapeutic delivery and enhanced wound management [16]. Owing to their versatility, tunable properties, and ability to mimic the ECM, hydrogels have emerged as one of the most promising platforms for advanced wound dressings and regenerative medicine applications.

### 3.2. Design Requirements of Hydrogels for Wound Healing Applications

**Figure 2:**
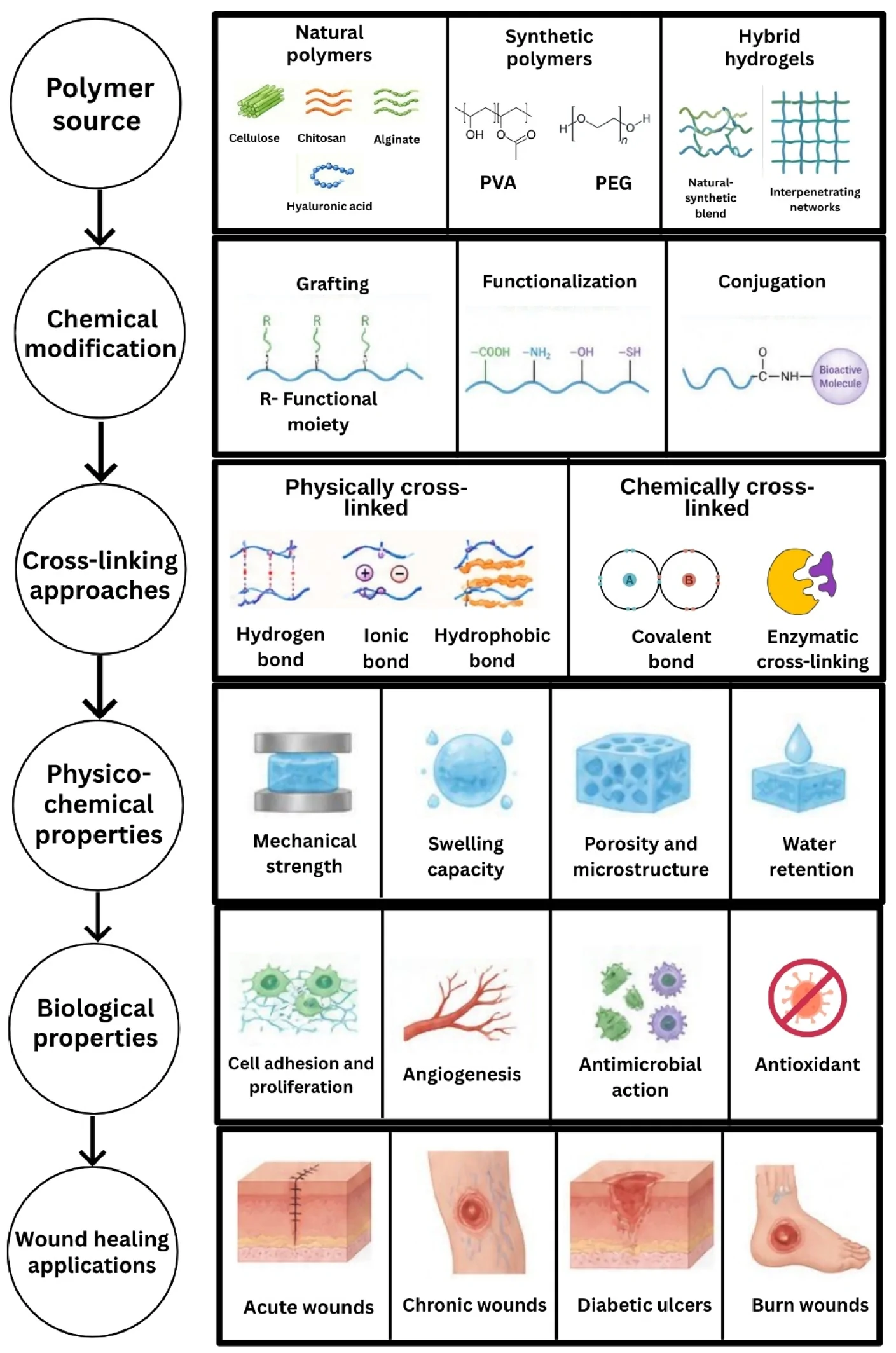
A comprehensive overview of the relationships among hydrogel polymer composition, chemical modification strategies, crosslinking approaches, resulting physicochemical and biological properties and their corresponding wound healing applications.

Hydrogels are considered ideal wound dressings with significant potential in skin regeneration and wound healing. They provide soothing effect, enhance patient compliance, maintain a moist environment, and facilitate softening of necrotic tissue [48]. Additionally, their non-adhesive nature allows for painless removal without damaging the wound. Hydrogels for wound healing should possess integrated physicochemical, mechanical, and biological properties that support tissue repair. They ideally resemble the ECM, offering moist three-dimensional atmosphere that fosters cell adhesion, migration, and proliferation while preserving moist wound environment for effective nutrition exchange and waste elimination [49]. They need to show mechanical compatibility, flexibility, and bioadhesiveness to guarantee appropriate stress distribution, adapt to uneven wounds, and stay localized at the application site. Biocompatibility and controlled biodegradability are essential to avoid unfavorable immunological reactions and promote the steady restoration of new tissue [14]. In addition, adequate porosity improves cell infiltration, angiogenesis, and transport processes. Novel hydrogels may provide controlled drug administration, oxygen permeability, and antibacterial activity, wherein stimuli-responsive systems provide targeted therapeutic activity, increasing their efficacy in wound healing purposes [50]. Recent years have seen growing interest in carrier-free hydrogel dressings. These systems offer intrinsic therapeutic benefits while minimizing issues such as batch variability and potential toxicity associated with carrier materials [51]. The interaction between hydrogel structure, biological effects and wound healing outcomes is illustrated in Figure 2.

## 4. Biopolymer-Based Hydrogels for Wound Healing Applications

Hydrogels for wound healing are broadly classified into synthetic polymer-based and natural biopolymer-based systems [52]. Synthetic hydrogels are commonly prepared from polymers such as PVA, PEG, PU, poly(vinyl pyrrolidone) (PVP), polyacrylamide (PAAm), and poly(N-isopropylacrylamide) (PNIPAAm) [53]. These polymers provide excellent mechanical strength, tunable physicochemical properties, reproducible synthesis, and controlled degradation. However, they are generally biologically inert and lack intrinsic antimicrobial, antioxidant, anti-inflammatory, and hemostatic activities, necessitating chemical modification or incorporation of bioactive agents to enhance tissue regeneration [54]. Furthermore, potential cytotoxicity associated with residual crosslinkers or degradation products, limited immunomodulatory activity, and, in some cases, poor biodegradability may hinder their long-term clinical application in wound healing.

Biopolymers play crucial role in wound healing applications due to their biocompatibility, biodegradability, non-toxicity, and ability to mimic the natural ECM [55]. These polymers provide moist wound environment, promote cell adhesion and proliferation, facilitate gas exchange, and protect the wound from microbial contamination, thereby accelerating the healing process. In addition, many biopolymers possess intrinsic bioactive properties such as antimicrobial, anti-inflammatory, and hemostatic effects, which further support tissue regeneration and reduce the risk of infection. Their versatile physicochemical characteristics also enable their fabrication into various wound dressing systems, including hydrogels, films, nanofibers, sponges, and scaffolds, suitable for both acute and chronic wound management [56]. Among the widely investigated biopolymers, cellulose and its derivatives, hyaluronic acid, sodium alginate, and chitosan have gained significant attention because of their excellent wound healing potential and ability to enhance tissue repair through different biological mechanisms [57].

### 4.1. Molecular Mechanisms and Targeted Design Strategies of Biopolymer Hydrogels for Acute and Chronic Wound Healing

Successful wound healing requires coordinated regulation of inflammation, oxidative stress, angiogenesis, ECM remodeling, and re-epithelialization. Biopolymer-based hydrogels not only provide a moist three-dimensional microenvironment that supports cell migration and tissue regeneration but also actively modulate multiple molecular pathways involved in wound repair. Although cellulose, chitosan, alginate, and hyaluronic acid possess distinct physicochemical characteristics, they converge on common biological mechanisms that collectively accelerate healing.

#### 4.1.1. Regulation of Inflammation

Persistent inflammation is a hallmark of chronic wounds, characterized by excessive production of pro-inflammatory cytokines, prolonged macrophage activation, and delayed tissue regeneration [44]. Biopolymer hydrogels help restore immune homeostasis by regulating inflammatory signaling pathways. Chitosan exhibits intrinsic immunomodulatory and antimicrobial activities, reducing the expression of TNF-α, IL-1β, and IL-6 while promoting anti-inflammatory mediators such as IL-10 [58]. Hyaluronic acid, particularly high-molecular-weight HA, suppresses excessive inflammatory responses by interacting with CD44 receptors and modulating NF-κB signaling [59,60]. Alginate hydrogels reduce inflammatory cell infiltration while maintaining a moist wound environment that minimizes secondary tissue damage [61]. Cellulose-based hydrogels primarily regulate inflammation indirectly by maintaining optimal hydration, reducing mechanical irritation, and serving as controlled-release platforms for anti-inflammatory therapeutics [62].

#### 4.1.2. Regulation of Oxidative Stress

Excessive ROS impair fibroblast function, damage cellular macromolecules, and delay wound healing. Biopolymer hydrogels alleviate oxidative stress by incorporating antioxidant molecules or stimulating endogenous antioxidant defenses. Chitosan and hyaluronic acid-based hydrogels have been shown to reduce intracellular ROS generation and enhance antioxidant enzymes, including superoxide dismutase (SOD) and catalase [63,64]. Cellulose and alginate hydrogels frequently serve as carriers for natural antioxidants such as curcumin, quercetin, epigallocatechin gallate, and cerium oxide nanoparticles, enabling sustained antioxidant release and activation of cytoprotective pathways such as Nrf2/HO-1 [65]. These strategies protect resident cells from oxidative injury and promote tissue regeneration.

#### 4.1.3. Promotion of Angiogenesis

Adequate neovascularization is essential for oxygen and nutrient delivery during wound repair. Biopolymer hydrogels enhance angiogenesis through sustained delivery of angiogenic factors, stem cell-derived extracellular vesicles (EVs), bioactive nanoparticles, and therapeutic cells. Increased expression of vascular endothelial growth factor (VEGF), basic fibroblast growth factor (bFGF), hypoxia-inducible factor-1α (HIF-1α), and activation of PI3K/AKT and ERK signaling pathways promote endothelial cell proliferation, migration, and new blood vessel formation [66]. Injectable, porous and self-healing hydrogel architectures further facilitate vascular infiltration, particularly in ischemic and diabetic wounds where impaired angiogenesis limits healing [67].

#### 4.1.4. Extracellular Matrix Remodeling and Re-Epithelialization

Effective wound healing requires balanced collagen synthesis and degradation. Biopolymer hydrogels stimulate fibroblast proliferation, collagen deposition, and keratinocyte migration while regulating MMPs and their tissue inhibitors (TIMPs) [55,68]. Activation of TGF-β/Smad signaling promotes collagen maturation and granulation tissue formation, whereas modulation of excessive MMP activity prevents ECM degradation commonly observed in chronic wounds. Hyaluronic acid enhances keratinocyte migration through CD44-mediated signaling, while chitosan stimulates fibroblast proliferation and collagen synthesis [69]. Cellulose- and alginate-based hydrogels provide structural support for cell attachment and facilitate sustained release of bioactive molecules that promote tissue remodeling and re-epithelialization [61].

#### 4.1.5. Targeted Hydrogel Design Strategies for Acute and Chronic Wounds

The pathological characteristics of acute and chronic wounds differ substantially, requiring tailored hydrogel design strategies. Acute wounds generally progress through normal healing phases and therefore benefit from hydrogels designed to maintain moisture, provide mechanical protection, achieve hemostasis, prevent microbial contamination, and support rapid re-epithelialization. In contrast, chronic wounds are characterized by persistent inflammation, bacterial biofilms, excessive oxidative stress, impaired angiogenesis, elevated protease activity, and defective ECM remodeling [70]. Accordingly, multifunctional hydrogels are increasingly engineered to combine antibacterial, antioxidant, anti-inflammatory, angiogenic, and immunomodulatory functions with controlled drug delivery and ECM-regulating capabilities.

Many multifunctional hydrogels promote macrophage polarization from the pro-inflammatory M1 phenotype to the pro-regenerative M2 phenotype, thereby reducing excessive inflammation and stimulating tissue repair through increased secretion of VEGF, TGF-β, and interleukin-10 (IL-10) [71]. In addition, hydrogels incorporating antioxidant molecules, enzymes, or nanozymes effectively scavenge excessive ROS, alleviating oxidative stress and protecting resident cells from damage, particularly in chronic diabetic wounds [72]. These effects enhance angiogenesis, promote ECM remodeling through regulated collagen deposition, and modulate cytokine expression by suppressing pro-inflammatory cytokines (TNF-α, IL-1β, and IL-6) while increasing anti-inflammatory mediators (IL-10 and TGF-β) [73]. Collectively, these immunomodulatory mechanisms create a favorable microenvironment that accelerates re-epithelialization, granulation tissue formation, and functional tissue regeneration. Recent advances, including injectable, self-healing, stimuli-responsive, nanocomposite, and extracellular vesicle-loaded hydrogels, enable dynamic adaptation to the wound microenvironment and provide sustained therapeutic effects [74,75]. Such rationally designed multifunctional hydrogel systems hold significant promise for improving the treatment of diabetic ulcers, pressure ulcers, venous leg ulcers, and other difficult-to-heal chronic wounds. Integrating different molecular mechanisms of biopolymers into rational hydrogel design is expected to accelerate the clinical translation of next-generation biopolymer-based wound dressings for both acute and chronic wound management.

### 4.2. Cellulose-Based Hydrogels

Cellulose is a natural polysaccharide found in plant cell walls and is made up of β-D-glucose units linked by β-(1→4) glycosidic bonds [76]. The cellulose molecule has long, linear, and unbranched structure in which adjacent glucose units are arranged in alternating manner, with each unit rotated 180° relative to the next. This arrangement allows the formation of extensive intra and intermolecular hydrogen bonds between hydroxyl groups present on neighboring chains.

Bacterial cellulose (BC), nanocellulose (NC), oxidized cellulose (OC), and cellulose nanocrystals (CNCs) are advanced forms of cellulose with unique structural and functional properties. These modified cellulose materials have excellent potential in wound healing applications due to their biocompatibility, biodegradability, high water absorption capacity, and mechanical strength. At the molecular level, cellulose-based hydrogels promote wound healing primarily by creating a favorable microenvironment that supports cellular activities essential for tissue repair. Cellulose-based hydrogels primarily facilitate tissue regeneration by providing structural support, efficient exudate management, and sustained delivery of bioactive molecules, thereby promoting fibroblast proliferation, collagen deposition, and re-epithelialization [77].

A comparative summary of their general characteristics and wound healing potential is presented in Table 2.

#### 4.2.1. Bacterial Cellulose (BC)

BC is a biosynthesized hydrogel produced by *Gluconacetobacter xylinus* through glucose polymerization by cellulose synthase [78]. The glucan chains are extruded through membrane complexes and self-assemble via hydrogen bonding into subfibrils, nanofibers, and finally ribbon-like structures. BC has gained significant attention in biomedical applications due to its hydrophilicity, biocompatibility, non-pyrogenicity, high wet strength, and transparency [79]. These properties have led to its use in commercial wound dressings (e.g., XCell^®^, Biofill^®^), primarily to support autolytic debridement. Its porous, crosslinked fiber network also enables the efficient incorporation of therapeutic agents, thereby enhancing its functionality in wound healing.

Various strategies have been employed to enhance the wound healing performance of BC hydrogels. Incorporation of bioactive compounds (e.g., curcumin inclusion complexes) improves moisture retention, sustained drug release, antioxidant and antibacterial activities, whereas structural modifications such as double-network and biofunctionalized BC hydrogels enhance adhesion, gelation, and hemostatic performance [80,81]. Similarly, composite BC hydrogels reinforced with polycaprolactone and loaded with antibiotics (gentamicin and streptomycin) provide controlled drug release, excellent cytocompatibility, and effective antimicrobial activity for infected wound management [82]. Overall, these studies demonstrate that functionalization of BC through bioactive incorporation, structural engineering, and composite design substantially enhances its physicochemical properties and therapeutic efficacy, establishing BC as a versatile platform for advanced wound dressing applications.

#### 4.2.2. Nanocellulose (NC)

NC is a nanosized fibrous material composed of β-(1→4)-linked D-glucose units with crystalline and amorphous regions. It possesses high surface area, high aspect ratio, strong hydrogen bonding, and excellent mechanical strength. NC is a strong, biodegradable, and renewable biomaterial available as bacterial NC, CNCs, and cellulose nanofibers [83]. It is widely used as a reinforcing agent to enhance the mechanical, thermal, and biological properties of polymeric systems. NC-based composites improve key features such as porosity, strength, moisture vapor transmission rate, and biocompatibility, making them highly suitable for tissue engineering and wound healing applications. Bacterial NC is widely regarded as ideal wound dressing material due to its chemical purity, excellent mechanical strength, and high water absorption capacity [84]. Bacterial NC promotes wound healing by enhancing epidermal and dermal regeneration, reducing inflammatory cell infiltration, and supporting fibroblast growth with low cytotoxicity, even without additional bioactive agents [85]. In burn wound models, bacterial NC accelerated healing compared to conventional gauze by enhancing tissue regeneration, angiogenesis, and collagen expression while reducing inflammation without causing systemic toxicity [86]. Crosslinking of NC enhances its structural stability, mechanical strength, and resistance to dissolution, while enabling tunable porosity and controlled drug release for improved wound healing performance.

Recent advances in NC-based hydrogels have focused on enhancing their functionality through drug delivery, injectability, and scaffold engineering. For controlled drug delivery, pH- and near-infrared-responsive NC hydrogels enabled on-demand tetracycline release while providing sustained antibacterial activity, favorable mechanical properties, and accelerated wound healing [87]. To overcome the inherent limitations of poor injectability and adhesion, functionalized NC reinforced with carboxymethyl chitosan (CMCS) produced injectable and adhesive hydrogels with enhanced cellular activity, conductivity, antibacterial efficacy, and improved in vivo wound repair [88]. Furthermore, NC-based 3D-printed scaffolds fabricated using dual crosslinking exhibited tunable mechanical properties and excellent biocompatibility, with increased stiffness promoting fibroblast proliferation, highlighting their potential for tissue engineering and wound regeneration [89]. Collectively, these strategies demonstrate that functional modification of NC significantly broadens its applicability in advanced wound healing through improved therapeutic delivery, handling characteristics, and structural support.

#### 4.2.3. Oxidized Cellulose (OC)

OC, particularly oxidized regenerated cellulose, is bioabsorbable hemostatic material extensively used in wound healing and surgical applications due to its excellent biocompatibility, biodegradability, and hemostatic properties. OC promotes rapid blood coagulation by absorbing blood and forming gelatinous matrix that enhances platelet adhesion and fibrin clot formation at the wound site. Additionally, its acidic nature contributes to mild antibacterial activity, thereby reducing the risk of infection [90]. The introduction of carboxyl groups during oxidation improves the fluid absorption capacity, swelling behavior, and biodegradability of cellulose, creating moist environment favorable for tissue regeneration, fibroblast proliferation, angiogenesis, and granulation tissue formation [77]. Owing to these properties, OC has been widely applied in chronic wounds, burns, trauma care, and surgical wound management in the form of gauzes, sponges, powders, and nonwoven dressings such as Surgicel^®^.

OC-based hydrogels have emerged as versatile platforms for both hemostasis and controlled drug delivery in wound healing. Sprayable OC nanofiber hydrogels exhibit superior hemostatic efficacy, rapidly controlling bleeding while promoting complete dermal and epidermal regeneration in vivo [91]. In addition, OC-based composite hydrogels have been successfully employed for the delivery of poorly soluble therapeutics, such as curcumin, where incorporation of solubilizing agents enhanced drug bioavailability, cellular uptake, tissue regeneration, collagen deposition, and wound closure [92]. These findings demonstrate that structural modification and hybridization of OC hydrogels significantly expand their therapeutic functionality by integrating rapid hemostasis with sustained drug delivery and enhanced tissue repair, although challenges such as rapid degradation and excessive swelling of composite systems remain.

#### 4.2.4. Cellulose Nanocrystals (CNCs)

CNCs are emerging as promising biomaterials for wound healing due to their high mechanical strength, large surface area, high aspect ratio, biodegradability, and excellent biocompatibility [93]. They can be incorporated into hydrogels, scaffolds, and composite dressings to maintain a moist environment, enhance cell adhesion and proliferation, and support tissue regeneration. Additionally, CNC-based systems enable controlled drug delivery and can be functionalized with antimicrobial agents to prevent infection and accelerate healing. Despite their potential, their clinical translation is restricted by issues including high production costs, scaling concerns, and concerns about sustainable biocompatibility [94].

CNCs have been widely employed as reinforcing nanomaterials to enhance the mechanical strength and therapeutic performance of hydrogel wound dressings. Functionalization with antimicrobial agents, including silver nanoparticles and polymyxin B, provides sustained or pH-responsive drug release and potent antibacterial activity against both Gram-positive *Staphylococcus aureus* (*S. aureus*) and Gram-negative *Escherichia coli* (*E. coli*) [95,96]. In addition to improving antimicrobial efficacy, these multifunctional CNC-based hydrogels promote collagen deposition, angiogenesis, tissue regeneration, and accelerated wound closure, highlighting the potential of CNCs as versatile reinforcing platforms for advanced wound healing applications.

#### 4.2.5. Cellulose Derivative-Based Hydrogels

**Figure 3:**
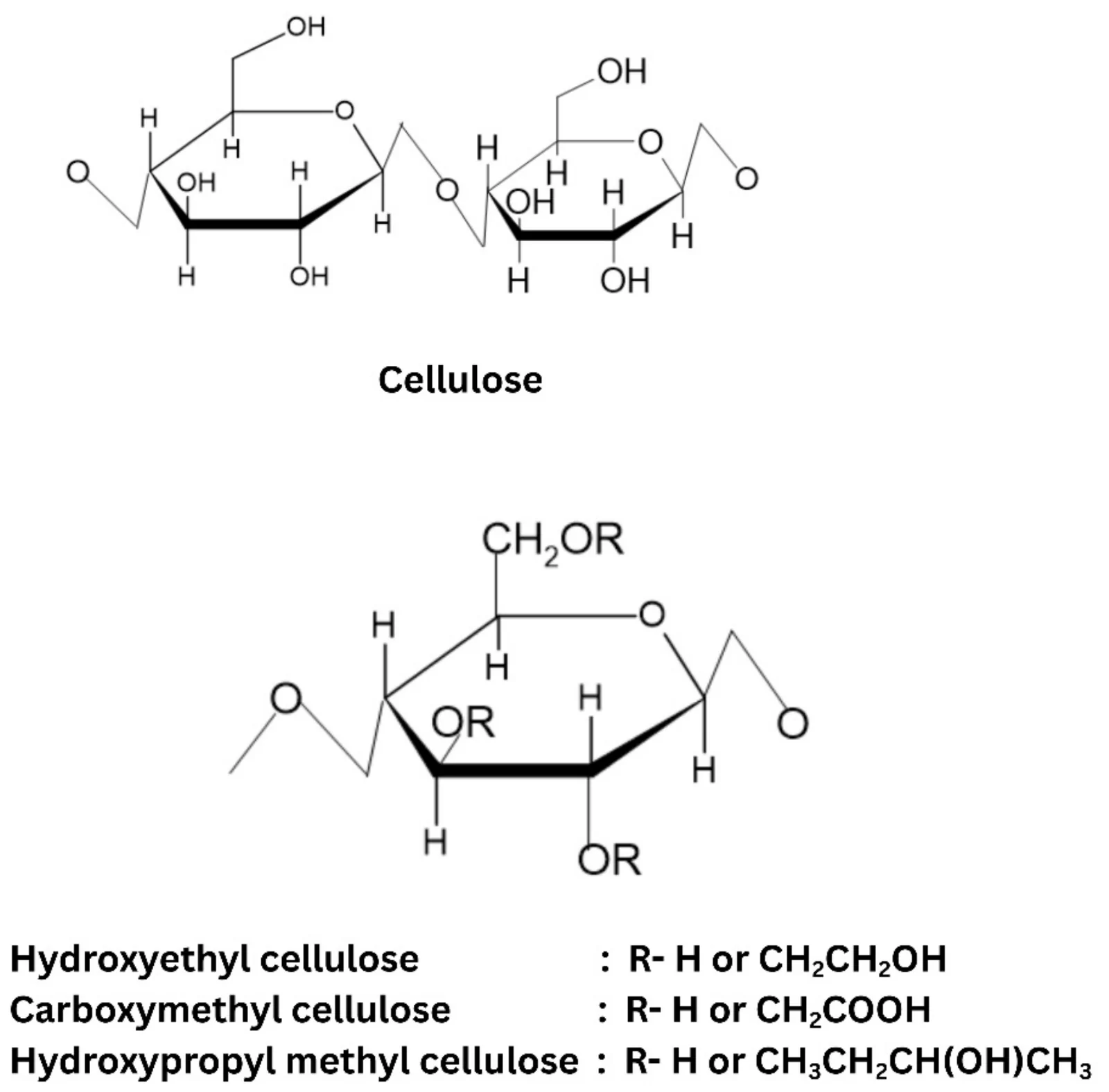
Chemical structure of native cellulose and its derivatives including carboxymethyl cellulose (CMC), hydroxyethyl cellulose (HEC) and hydroxypropyl methyl cellulose (HPMC).

Cellulose derivatives such as carboxymethyl cellulose (CMC), hydroxyethyl cellulose (HEC), and hydroxypropyl methylcellulose (HPMC) are widely used in wound healing applications because of their biocompatibility, biodegradability, non-toxicity, and excellent film-forming and moisture-retention properties. The chemical structure of cellulose and its derivatives are illustrated in Figure 3.

#### 4.2.6. Carboxymethyl Cellulose (CMC)-Based Hydrogels

CMC is a water-soluble anionic derivative of cellulose produced by the chemical substitution of hydroxyl groups (-OH) present in the cellulose backbone with carboxymethyl groups (-CH_2_COOH). Structurally, CMC consists of β-(1→4)-linked D-glucose units similar to native cellulose, but the introduction of carboxymethyl substituents imparts improved hydrophilicity, solubility, swelling capacity, and viscosity [97]. The presence of negatively charged carboxylate groups provides CMC with polyelectrolyte behavior, enabling strong water absorption, gel formation, mucoadhesion, and interactions with biological molecules. Additionally, the polymer exhibits excellent film-forming ability, biocompatibility, biodegradability and mechanical flexibility, making it highly suitable for wound dressings, drug delivery systems, hydrogels, and tissue engineering applications [98]. The amorphous nature introduced by carboxymethyl substitution also contributes to enhanced water dispersibility and reduced crystallinity compared to native cellulose. CMC-based wound dressings are widely used due to their biocompatibility, biodegradability, low toxicity, and cost-effectiveness [99]. CMC-based hydrogels exhibit desirable properties such as high swelling capacity, moisture retention, and good mechanical stability, which are essential for effective wound healing. CMC-based hydrogels incorporating diverse active ingredients for wound healing are highlighted in Table 3.

Natural bioactive agents have been extensively incorporated into CMC-based hydrogels to enhance wound healing through their intrinsic antioxidant, antibacterial, and anti-inflammatory properties. Grapefruit seed extract-loaded CMC/HPMC hydrogels demonstrated excellent antimicrobial activity, improved flexibility, and stable nanoparticle formation, highlighting their potential as natural alternatives to synthetic drug-loaded dressings [108]. Similarly, phytic acid-crosslinked CMC hydrogels exhibited enhanced stability, controlled drug release, antioxidant activity, and good biocompatibility, while effectively inhibiting both *Escherichia coli* (*E. coli*) and *Staphylococcus aureus* (*S. aureus*) [109]. Incorporation of sericin further improved the multifunctionality of CMC hydrogels by reducing oxidative stress, inflammation, and microbial infection, while promoting collagen deposition, skin appendage formation, and tissue regeneration in diabetic wounds through modulation of oxidative stress markers and inflammatory mediators [110]. Collectively, these studies demonstrate that natural bioactive agents not only provide direct therapeutic effects but also improve the biological performance of CMC-based hydrogels for chronic wound management.

Inorganic nanoparticles and synthetic therapeutics have further expanded the capabilities of CMC hydrogels by improving their physicochemical properties and therapeutic efficacy. Zinc oxide nanoparticles and nanostructures significantly enhanced the mechanical strength, swelling behavior, water vapor transmission, and antibacterial activity of CMC-based hydrogels against *S. aureus* and *E. coli*, while simultaneously promoting fibroblast adhesion, proliferation, and accelerated wound healing in vivo [111,112,113]. In parallel, incorporation of the synthetic therapeutic agent simvastatin provided sustained drug release and effective immunomodulation by suppressing pro-inflammatory cytokines (IL-6 and TNF-α) and increasing anti-inflammatory mediators (IL-10 and TGF-β), thereby facilitating angiogenesis, tissue regeneration, and chronic wound repair [114,115]. These findings highlight the complementary roles of nanomaterials and synthetic drugs in enhancing both the structural integrity and biological activity of CMC-based hydrogels.

Recent developments have focused on multifunctional regenerative hydrogels that integrate self-healing behavior, pH responsiveness, and controlled therapeutic delivery to address the complex wound microenvironment [99,116]. Among these, hydrogels incorporating adipose-derived stem cell exosomes have shown remarkable regenerative potential by promoting angiogenesis, neovascularization, and tissue remodeling while exhibiting antibacterial and anti-inflammatory activities [117]. Similarly, self-healing hydrogels based on dialdehyde CMC and carboxyethyl chitosan provided sustained exosome delivery, restored tissue morphology and function, and significantly accelerated diabetic wound healing [118]. Overall, these advances demonstrate that combining CMC with natural therapeutics, inorganic nanomaterials, synthetic drugs, and stem cell-derived products offers a versatile strategy for developing next-generation multifunctional wound dressings with enhanced regenerative efficacy.

#### 4.2.7. CMC-Based Composite Hydrogels

The combination of different polymers to form composite hydrogels is commonly used to improve the mechanical strength, biocompatibility, and functional properties of wound healing biomaterials. Hydrogel films containing low methoxyl pectin, gelatin, and CMC demonstrated enhanced mechanical stability, water retention, fluid uptake, and water-vapour transmission rate [119].

**Figure 4:**
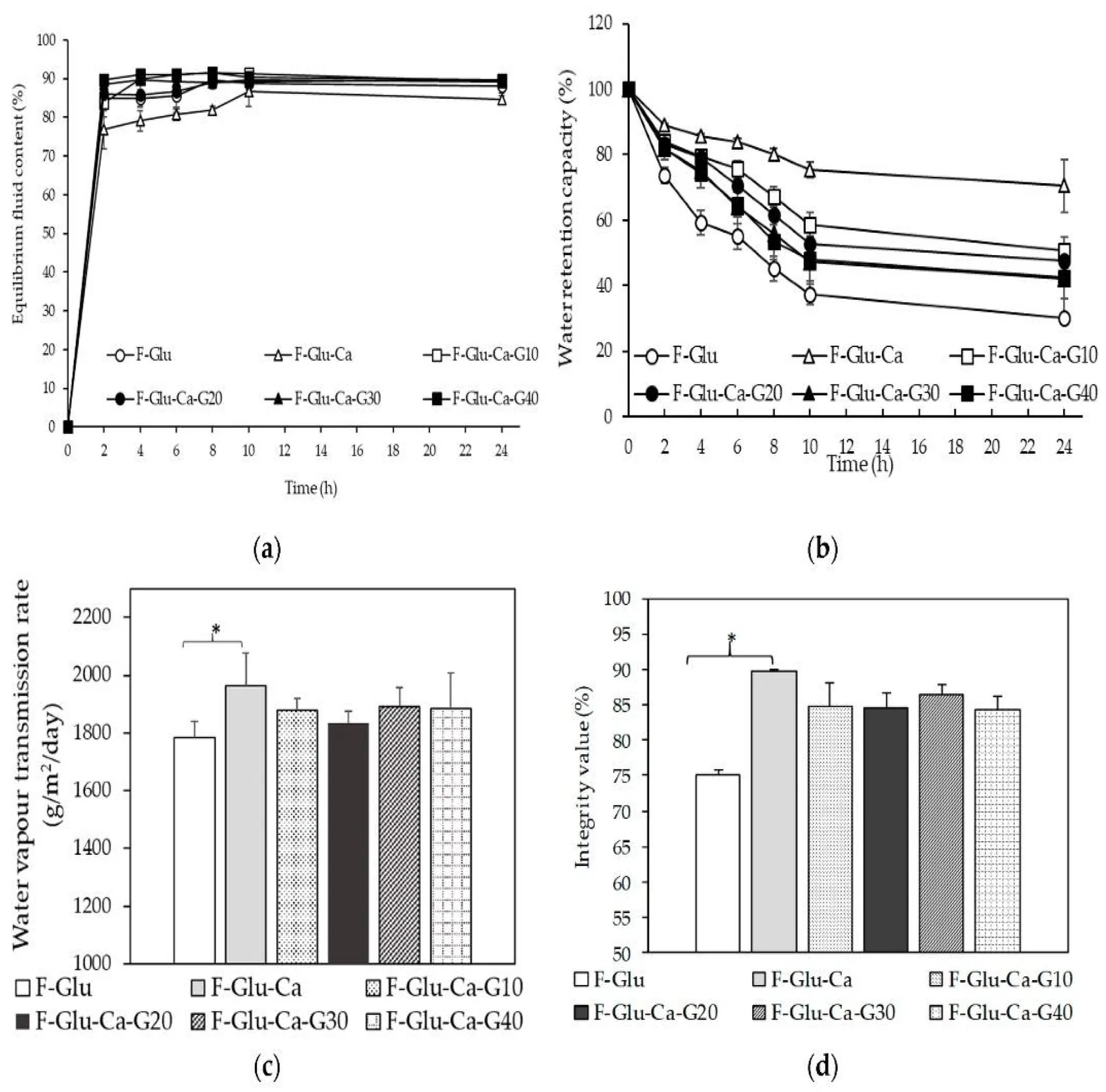
Fluid uptake ability (**a**), water retention capacity (**b**), water vapor transmission rate (**c**), and integrity value (**d**) of F-Glu, F-Glu-Ca, F-Glu-Ca-G10, F-Glu-Ca-G20, and F-Glu-Ca-G30 hydrogel films. Statistical significance was considered at * *p* < 0.05. Reproduced from Ref. [119].

Composite CMC-based hydrogels have been extensively developed to enhance the mechanical stability, antibacterial efficacy, and regenerative performance of wound dressings. As shown in Figure 4, incorporation of glycerin and dual crosslinking with glutaraldehyde and calcium chloride significantly improved fluid absorption, moisture retention, water vapor transmission, and film integrity, with the F-Glu-Ca-G30 formulation demonstrating the most balanced physicochemical and functional properties [119]. Furthermore, incorporation of povidone iodine imparted potent antibacterial activity against *Staphylococcus aureus* (*S. aureus*). Functionalization with carbon quantum dots further expanded the therapeutic potential of composite hydrogels by providing photothermal antibacterial activity against *S. aureus* and *Escherichia coli* (*E. coli*), promoting skin regeneration, and enabling pH-responsive wound monitoring [120,121]. Advances in 3D printing have also enabled the fabrication of patient-specific CMC-based hydrogels with controlled architectures, enhanced mechanical properties, antioxidant activity, and improved angiogenesis and wound healing [122,123]. Similarly, recombinant human collagen–CMC composite hydrogels improved gelation, mechanical strength, hemostatic activity, stem cell proliferation, fibroblast migration, and tissue regeneration, demonstrating the ability of composite formulations to integrate structural reinforcement with enhanced biological performance [124]. Overall, composite CMC-based hydrogels represent promising multifunctional wound dressings, although challenges related to large-scale manufacturing and clinical translation remain.

#### 4.2.8. Hydroxyethyl Cellulose (HEC)-Based Hydrogels

HEC is a nonionic, water-soluble cellulose ether synthesized through the reaction of cellulose with ethylene oxide, resulting in the substitution of hydroxyl groups in the cellulose backbone with hydroxyethyl groups (–CH_2_CH_2_OH). Structurally, HEC retains the β-(1→4)-linked D-glucose units of native cellulose while exhibiting reduced crystallinity and enhanced hydrophilicity due to hydroxyethyl substitution [125]. The degree of substitution and molar substitution significantly influence its physicochemical properties, including solubility, viscosity, swelling behavior, and film-forming ability. In hydrogel systems, HEC promotes high water diffusion and porous structures, thereby supporting tissue regeneration and enabling nanoparticle incorporation [126]. Oxidation of HEC (e.g., using sodium periodate) produces oxidized HEC with reactive aldehyde groups, which can form hydrogels with suitable gelation properties, high swelling capacity, good water retention, and mechanical stability, making them ideal for wound healing applications.

Natural products have been extensively incorporated into HEC-based hydrogels to enhance wound healing through their intrinsic antibacterial, antioxidant, and anti-inflammatory activities. Hydrogels containing extracts of *Satureja montana*, *Salvia sclarea*, *Coriandrum sativum*, and *Lavandula angustifolia* effectively promoted epidermal proliferation and accelerated healing of *Pseudomonas aeruginosa* (*P. aeruginosa*)-infected burn wounds [127]. Similarly, curcumin-loaded HEC/PVA biocomposite hydrogels derived from sugarcane bagasse cellulose exhibited excellent mechanical strength, swelling capacity, antioxidant and antibacterial properties, as well as enhanced fibroblast compatibility, collagen deposition, reduced inflammation, and approximately 95% wound closure within 12 days [128]. These findings demonstrate that natural therapeutics not only improve the biological activity of HEC hydrogels but also contribute to sustainable wound dressing development.

Antimicrobial nanomaterials have further expanded the functionality of HEC-based hydrogels by improving their physicochemical properties and infection control. Selenium nanoparticle-loaded oxidized HEC nanohydrogels exhibited strong antioxidant, antimicrobial, and regenerative activities, achieving complete wound closure in vitro within 12 h and nearly complete wound healing in vivo by day 14 [129]. Likewise, HEC-guided hydrogels incorporating silver nanoparticles or tungsten oxide demonstrated excellent biocompatibility, broad-spectrum antibacterial activity against methicillin-resistant *Staphylococcus aureus* (MRSA), *Salmonella* spp., and *P. aeruginosa*, together with antibiofilm activity, enhanced structural stability, and accelerated wound healing [126,130].

Peptide-based self-assembled hydrogels represent another promising therapeutic strategy for infected wounds. pH-tolerant self-assembling peptide hydrogels loaded with the phage-derived antimicrobial endolysin LysSYL or dual-drug Nap-GFFKH systems exhibited potent antibacterial activity, eliminating MRSA while accelerating wound closure by up to 95.1% within 14 days [131]. Collectively, these studies demonstrate that integrating natural therapeutics, antimicrobial nanomaterials, and self-assembling peptide systems into HEC-based hydrogels substantially enhances antimicrobial efficacy, tissue regeneration, and wound healing, highlighting their potential as multifunctional wound dressings for chronic and infected wounds.

#### 4.2.9. HEC-Based Composite Hydrogels

Composite hydrogels made from various polymers are gaining popularity in wound healing applications owing to their complementing physicochemical and biological characteristics. Oxidized HEC/CMCS composite hydrogels developed by Schiff-base reactions showed adequate gelation, swelling, water retention, and excellent biocompatibility, suggesting their potential as wound dressings. Additionally, injectable quaternized HEC/mesocellular silica foam hydrogel sponges were formulated for antibacterial activity and effective bleeding control [132]. These hydrogels showed rapid expansion, good absorbency, enhanced blood clotting, and rapid wound healing capabilities. Interpenetrating polymer networks are important hydrogel structures commonly formed through covalent crosslinking; however, residual reactive groups may cause toxicity and compromise protein stability [133]. To address these limitations, non-covalent crosslinking strategies based on hydrogen bonding between carboxyl (–COOH) and hydroxyl (–OH) groups have been explored to improve biocompatibility and reduce toxicity [134].

Interpenetrating polymer networks and hydrogen-bonded composites with CMCS improve porosity, swelling behavior, sustained growth factor release, and wound healing through enhanced re-epithelialization and angiogenesis [135]. Incorporation of functional additives, including tannic acid, bacterial cellulose, and iron (III) ions, further enhances mechanical strength, antioxidant and antibacterial activities against *Escherichia coli* (*E. coli*) and *Staphylococcus aureus* (*S. aureus*), while imparting self-healing and conductive properties suitable for advanced wound dressings and biosensing applications [136,137]. Similarly, regenerated cellulose-based HEC composites exhibit excellent water absorption, moisture retention, and exudate management, providing enhanced mechanical stability and a favorable microenvironment for wound repair [138]. Collectively, these composite design approaches demonstrate that combining HEC with complementary polymers, bioactive molecules, and functional nanomaterials significantly broadens its applicability for developing multifunctional wound dressings with improved structural integrity and therapeutic efficacy. Table 4 outlines HEC-based formulations, including their composition, functions, mechanistic outcomes, design strategy, and translational stage.

#### 4.2.10. Hydroxypropyl Methylcellulose (HPMC)-Based Hydrogels

HPMC or hypromellose is a semi-synthetic, non-ionic cellulose ether derived from natural cellulose, consisting of β-(1→4)-linked D-glucose cellulose backbone with methoxy and hydroxypropyl substitutions. These chemical modifications enhance its water solubility, viscosity, flexibility, film-forming ability, and gel-forming properties. The hydroxypropyl groups improve hydration and swelling, whereas methoxy groups contribute to thermal stability and gel formation. The physicochemical properties of HPMC, including rheology, crystallinity, and hydrophilic/hydrophobic behavior, are influenced by the degree of substitution and molar substitution [147]. Due to the presence of both polar and non-polar groups, HPMC can interact with various materials through intermolecular and intramolecular interactions. However, being non-ionic, it exhibits limited adhesion. HPMC also demonstrates thermoreversible sol–gel transition above 60 °C, ensuring stability under physiological conditions. HPMC is extensively used in hydrogel wound dressings because of its excellent physicochemical and biological properties. It readily absorbs water and swells to form stable hydrogels that maintain a moist environment favorable for wound healing [148]. Its good viscosity, viscoelasticity, and bioadhesive nature enhance application and retention at the wound site. The bioadhesive property of HPMC is attributed to the presence of hydroxyl (–OH) groups that facilitate hydrogen bonding with water molecules and adjacent polymer chains [149]. HPMC is also biocompatible, non-toxic, and non-irritant, making it suitable for direct contact with wounded tissue. Additionally, its film-forming ability produce flexible protective barrier against microbial contamination and mechanical injury while improving the tensile strength, adhesion, and flexibility of hydrogels. HPMC further exhibits high water retention capacity and semi-permeable characteristics that support oxygen diffusion, moisture balance, and autolytic debridement, all of which are essential for effective wound healing. Moreover, HPMC-based hydrogels provide controlled drug release, promote cell proliferation and tissue regeneration, and enhance wound healing efficiency, making them promising materials for advanced wound dressing applications.

HPMC-based formulations have been utilized for delivering various bioactive natural agents in wound healing applications. Collagen is a biodegradable, biocompatible, and non-toxic protein widely used in wound healing and drug delivery applications [150]. Fish-derived collagen has emerged as a promising alternative to human collagen because of its favorable biological properties [151]. Curcumin-loaded fish scale collagen/HPMC nanogels demonstrated prolonged drug release and enhanced wound healing efficacy [152]. Multifunctional HPMC-based composites containing *Eucalyptus camaldulensis* extracts exhibited strong antimicrobial, antioxidant, anti-inflammatory, and hemostatic activities, along with enhanced cell migration and good biocompatibility [153]. Stimuli-responsive hydrogels capable of responding to environmental triggers such as pH, temperature, and moisture have gained attention for controlled and targeted drug delivery in wound healing. An acacia honey and glycyrrhizic acid-loaded poloxamer-HPMC hydrogel exhibited favorable gelation behavior, effective exudate absorption, antimicrobial and antioxidant activities, and accelerated tissue regeneration, resulting in complete wound closure within 15 days [154].

**Figure 5:**
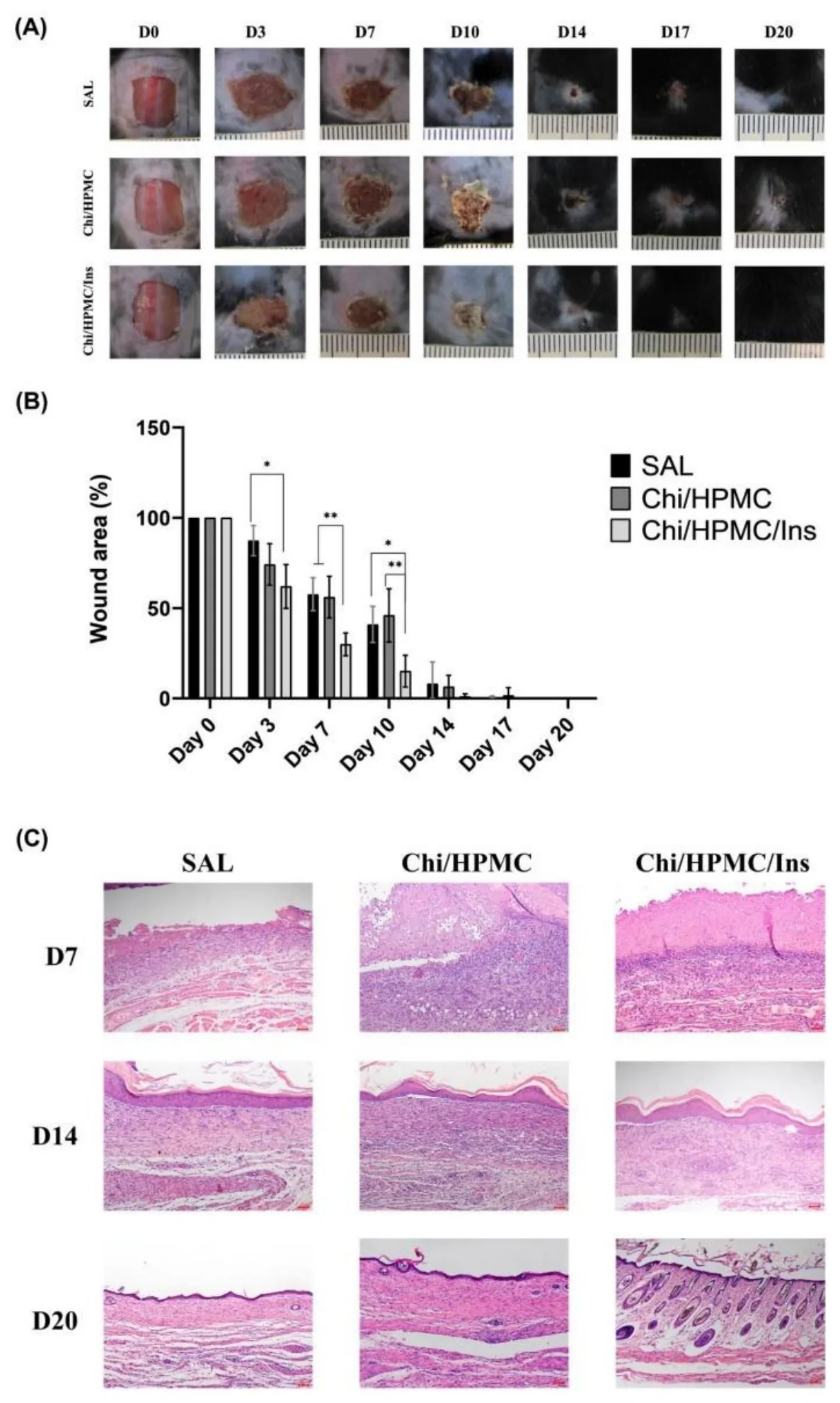
(**A**) Representative images showing wound contraction in saline (SAL), chitosan/HPMC (Chi/HPMC) hydrogel, and insulin-loaded chitosan/HPMC (Chi/HPMC/Ins) hydrogel treated groups on days 0, 3, 7, 10, 14, 17, and 20 post-injuries. (**B**) The percentage of wound contraction for every treatment group is presented as mean ± SD, with * *p* < 0.05 and ** *p* < 0.01 indicating statistical significance. (**C**) Typical histological sections of wound tissues on days 7, 14, and 20 after injury, showing the progress of healing and tissue regeneration. Reproduced from Ref. [158].

On the other hand, HPMC-based formulations are also utilized for delivering various synthetic therapeutics in wound healing applications. Corneal alkali burns are severe ophthalmic injuries with limited treatment options [155]. Diclofenac-loaded methylcellulose/HPMC thermosensitive gels demonstrated temperature-responsive gelation, sustained drug release, anti-inflammatory effects, and improved healing in corneal alkali burn models [156]. Topical insulin has been shown to enhance wound healing by promoting keratinocyte migration, angiogenesis, and tissue regeneration through activation of IR/IRS-PI3K-AKT signaling pathways [157]. Insulin has been widely investigated in wound healing due to its ability to promote cell proliferation, angiogenesis, collagen synthesis, and tissue regeneration, particularly in chronic and diabetic wounds. The wound healing efficacy of saline (negative control), chitosan/HPMC hydrogel, and insulin-loaded chitosan/HPMC hydrogel was evaluated in full thickness diabetic mouse wound model over 20 days [158]. The insulin-loaded hydrogel group demonstrated significantly greater wound contraction on days 3, 7, and 10 compared to the negative control group, and on days 7 and 10 compared to the chitosan/HPMC hydrogel group. Histological evaluation on days 7, 14, and 20 revealed improved granulation tissue organization, reduced scar formation, and hair follicle regeneration in the insulin treated group, indicating enhanced wound healing and tissue regeneration potential (Figure 5).

#### 4.2.11. HPMC-Based Composite Hydrogels

Crosslinking agents are used to connect polymer chains through covalent or non-covalent bonds, forming stable three-dimensional hydrogel networks [159]. Among these agents, citric acid has attracted considerable interest because of its low cost and non-toxic nature [160]. Citric acid-crosslinked composite hydrogel films composed of sodium CMC and HPMC exhibited reduced swelling, pore size, and tensile strength, along with increased glass transition temperature and flexibility. These films also demonstrated nanoporous architecture, sustained drug release, antibacterial activity, and promising wound healing potential [161]. Hydrogel films combining HPMC or hydroxypropyl cellulose (HPC) with sodium alginate loaded with gatifloxacin demonstrated improved mechanical strength, flexibility, moisture regulation, and sustained antibiotic release for up to 48 h, while also preventing microbial penetration [162]. The incorporation of HPMC contributed to controlled drug release and the maintenance of optimal wound environment. Antimicrobial photodynamic inactivation is an effective strategy against drug-resistant infections in which light-activated photosensitizers generate ROS that destroy microbial cells without inducing resistance [163]. The incorporation of HPMC improves viscosity, mechanical strength, and bioadhesion, thereby prolonging retention at the wound site and enhancing therapeutic efficacy. Although excessive concentrations of HPMC may reduce drug diffusion, optimized formulations provide improved stability and bactericidal activity suitable for wound healing applications. Furthermore, HPMC containing chitosan composites demonstrated improved flexibility, porosity, absorption capacity, platelet and erythrocyte adhesion, and enhanced hemostatic efficacy [164]. Composite hydrogels composed of collagen, PVA, and HPMC have emerged as promising wound dressings due to their excellent biocompatibility, antibacterial activity, and self-healing properties [41]. Crosslinked with borax and incorporated with polyhexamethylene biguanide as antibacterial agent, these multifunctional hydrogels demonstrated strong adaptability to wounds, effective antibacterial action against *E. coli* and *S. aureus*, and enhanced wound healing with accelerated collagen deposition. Binary hydrogels composed of two polymers are widely used in wound healing dressings because of their enhanced physicochemical and biological properties. Since pure polyvinylpyrrolidone hydrogels exhibit low swelling and poor mechanical strength, they are often combined with HPMC to improve physicomechanical performance [165]. In these systems, HPMC mainly governs gel properties, while polyvinylpyrrolidone enhances bioadhesiveness without significantly affecting viscosity or rheological behavior [149].

Hemostatic materials are essential for rapid bleeding control in trauma and injury management. High fluid absorption and coagulation-promoting ability are important characteristics of effective hemostatic materials. In a recent study, medicinal plant extracts with proven hemostatic and antibacterial properties such as *Salvadora persica* (Miswak), *Achillea millefolium* L. (Yarrow), *Capsella bursa-pastoris* Medik. (shepherd’s purse), and *Equisetum arvense* L. (horsetail) were incorporated into superabsorbent dressings prepared from HPMC, agar-agar, and carbopol matrices [166]. These composite dressings exhibited enhanced swelling capacity, cytocompatibility, hemostatic efficiency, and antibacterial activity. In vivo studies further confirmed their effectiveness in controlling bleeding and promoting wound healing, highlighting their potential as multifunctional wound dressings. Plant extracts such as henna (*Lawsonia inermis* L.) and chamomile (*Matricaria chamomilla* L.) contribute antibacterial and antioxidant effects due to bioactive compounds like terpenoids, phenolics, tannins, and flavonoids. When incorporated into HPMC/CMC hydrogels, these systems enable controlled release of active agents, enhancing antimicrobial activity and promoting efficient wound healing [167].

HPMC-based hydrogels containing synthetic or multifunctional therapeutic agents provide controlled and sustained drug delivery for improved wound healing outcomes [148]. HPMC/polyvinylpyrrolidone hydrogels loaded with silver nanoparticles exhibited enhanced antibacterial activity against *E. coli* and *S. aureus* due to improved nanoparticle stabilization and dispersion [168]. Similar superporous polyacrylamide/HPMC hydrogels incorporating silver nanoparticles demonstrated controlled silver release, effective antibacterial activity, rapid fluid absorption, and improved healing of infected wounds with reduced scar formation [169].

Recent advances include multifunctional composite hydrogels containing nanoparticles, therapeutic agents, and stimuli-responsive systems that enhance antibacterial activity, hemostatic performance, and tissue regeneration [148]. Advanced fabrication techniques such as electrospinning and 3D printing have further improved the mechanical and biological performance of these hydrogels [170]. Smart HPMC-based hydrogels responsive to pH, temperature, and moisture are also gaining attention for personalized wound management, although further clinical studies are needed for successful large-scale application [41]. Overall, HPMC-based hydrogels represent promising multifunctional materials for the management of acute and chronic wounds, with strong potential for advanced wound care and future clinical applications. Table 5 presents HPMC-based formulations, highlighting their composition, functions, mechanistic outcomes, design strategy, and translational stage.

### 4.3. Chitosan-Based Hydrogels

Chitosan is a natural cationic polysaccharide obtained by the partial deacetylation of chitin, consisting mainly of β-(1→4)-linked D-glucosamine and N-acetyl-D-glucosamine units. Its chemical structure contains reactive amino (–NH_2_) and hydroxyl (–OH) groups, which enable extensive chemical modification and crosslinking for hydrogel formation. Chitosan properties vary with source, including degree of deacetylation, molecular weight, and crystallinity. Higher deacetylation increases deacetylated units, improving solubility, charge density, and biocompatibility. Molecular weight affects viscosity and mechanical characteristics, whereas amorphous or crystalline forms impact barrier function and strength [178]. The building block of chitosan provides specific qualities such as biodegradability, structural stability, hydrophilicity, gel-forming capabilities, and the capacity to accomplish controlled release of drugs [179].

At the molecular level, chitosan promotes wound healing by modulating multiple signaling pathways involved in inflammation, oxidative stress, angiogenesis, and tissue regeneration. Beyond its intrinsic antibacterial activity, chitosan exerts significant anti-inflammatory effects by modulating both innate immune responses and macrophage function [180,181]. Chitosan promotes macrophage polarization from the pro-inflammatory M1 phenotype toward the pro-regenerative M2 phenotype, thereby reducing the production of pro-inflammatory cytokines, including TNF-α, IL-1β, and IL-6, while enhancing the secretion of anti-inflammatory mediators such as IL-10 and TGF-β [182]. In addition, chitosan suppresses activation of the nuclear factor-κB (NF-κB) signaling pathway, decreases oxidative stress, and limits excessive inflammatory cell infiltration, thereby facilitating angiogenesis, collagen deposition, and tissue remodeling during wound repair [183].

Natural therapeutic agents like flavonoids, phenolic acids, and essential oils incorporated into chitosan-based hydrogels have shown significant potential in wound healing due to their antioxidant, anti-inflammatory, and antimicrobial properties. Chitosan hydrogels loaded with apigenin enhanced diabetic wound healing through improved antioxidant activity and moisture retention [184], while taxifolin-loaded thermo-responsive hydrogels provided controlled drug release and accelerated skin repair [185]. Injectable hydrogels containing protocatechuic acid demonstrated strong antimicrobial and antioxidant effects, significantly enhancing collagen deposition and wound healing in vivo [186]. Similarly, essential oil-loaded chitosan composite hydrogels, particularly those containing eucalyptus oil, exhibited potent antibacterial activity, reduced inflammation, promoted growth factor expression, and accelerated burn wound healing [187]. Quaternized chitosan (QCS)-based hydrogels incorporating natural agents such as catechol and tannic acid have demonstrated excellent wound healing potential due to their thermosensitive, injectable, adhesive, antibacterial, antioxidant, and self-healing properties. Catechol-modified QCS hydrogels enhanced tissue adhesion and angiogenesis [188], while tannic acid-containing QCS hydrogels promoted ROS scavenging, coagulation, collagen deposition, and accelerated diabetic wound healing [189]. Incorporation of protamine further improved antibacterial activity, angiogenesis, adhesion, and tissue regeneration in periodontal wound healing [190]. Alkylated chitosan-based systems incorporating phytochemicals have shown promising wound healing potential due to improved drug loading, controlled release, and enhanced antioxidant, anti-inflammatory, and antimicrobial activities. These composite formulations promote faster wound closure, reduce infection and inflammation, and support tissue regeneration through the intrinsic hemostatic and biocompatible properties of alkylated chitosan [191].

Chitosan-based hydrogels can be loaded with variety of therapeutic agents, including bacteriostatic compounds, antibiotics, bacteriophages, and other bioactive substances. This incorporation enhances their antimicrobial effectiveness, allowing efficient inhibition of pathogens, lowering the risk of infection, and improving the treatment of chronic wounds. Chitosan hydrogels loaded with doxycycline demonstrated effective antibacterial activity against *S. aureus* and *E. coli* [192]. Chitosan enhances photodynamic inactivation activity against bacteria, fungi, and biofilms [193]. CMCS-based hydrogels have demonstrated excellent potential for wound healing due to their injectable, self-healing, biodegradable, and biocompatible properties.

Injectable CMCS hydrogels crosslinked with Fe^3+^ and Al^3+^ ions exhibited rapid gelation, self-healing behavior, and thermoresponsive properties suitable for wound dressing applications [194]. Genipin-crosslinked CMCS sponges showed superior absorption, hemostatic activity, and tissue regeneration compared to hydrogel and membrane forms [195], whereas poly(ethylene glycol) diacrylate (PEGDA)-crosslinked CMCS hydrogels reduced inflammation and promoted collagen formation and epidermalization without cytotoxicity [196]. In addition, trimethyl chitosan-povidone-iodine formulations demonstrated enhanced antimicrobial activity against multidrug-resistant pathogens and accelerated wound healing through improved collagen formation and re-epithelialization [197]. QCS-based hydrogels incorporating synthetic components have demonstrated excellent wound healing potential due to their thermosensitive, injectable, adhesive, antibacterial, and self-healing properties. Thermosensitive QCS/F127 hydrogels showed rapid gelation, sustained drug release, antibacterial activity, and strong hemostatic performance [198], while QCS hydrogels containing silver nanoparticles significantly enhanced antibacterial efficacy and accelerated burn wound healing compared with conventional silver dressings [199].

**Figure 6:**
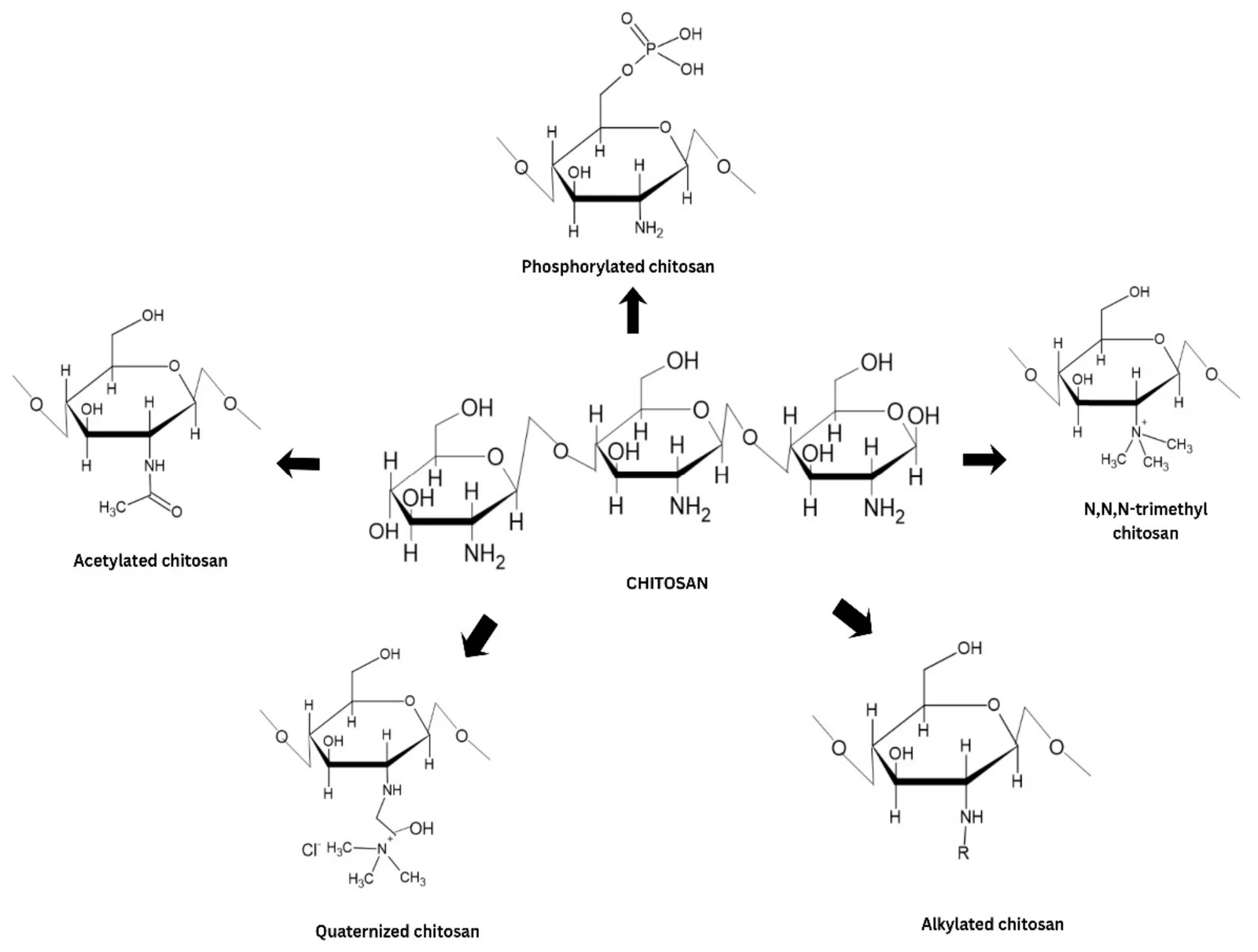
Chemical structure of chitosan and its derivatives.

Peptide-loaded chitosan hydrogels have shown strong potential in wound healing due to their antimicrobial activity, biocompatibility, and ability to promote tissue regeneration. Chitosan hydrogels loaded with tilapia-derived peptides exhibited strong antibacterial activity against *E. coli* and *S. aureus* while enhancing skin regeneration and burn wound healing [200]. Similarly, collagen peptide-modified chitosan hydrogels demonstrated improved antioxidant activity, excellent biocompatibility, and enhanced fibroblast proliferation [201]. Thermoresponsive chitosan hydrogels conjugated with recombinant human collagen peptides further improved mechanical strength, angiogenesis, cell infiltration, and accelerated healing of second-degree burn wounds [202]. A chitosan-based hydrogel co-loaded with hydrogen peroxide and antimicrobial peptides demonstrated synergistic antibacterial and antibiofilm activity against MRSA, significantly improving wound healing and wound closure in vivo. The combined system enhanced antimicrobial efficacy compared to hydrogen peroxide alone, highlighting its potential for treating infected and biofilm-associated wounds [203]. The chemical structure of chitosan and its derivatives are shown in Figure 6.

Recent advances in smart chitosan-based hydrogels include the incorporation of metal nanoparticles and stimuli-responsive systems (pH, thermo, and photosensitive), enhancing antimicrobial activity and wound healing efficacy. Self-healing and drug-loaded hydrogels further improve functionality and durability. For successful clinical and industrial application, problems including inadequate adhesion, controlled drug release, biosafety concerns, and large-scale manufacturing must be addressed.

#### 4.3.1. Chitosan-Based Composite Hydrogels

Chitosan-based hydrogels function as advanced dressings in wound healing by developing three-dimensional, hydrated network that simulates the ECM, providing moist environment, facilitating cell proliferation, and supporting controlled delivery of drugs [204]. Additionally, they have hemostatic, antibacterial, and bioadhesive features that support multiple stages of healing, including tissue regeneration, inflammation control, and hemostasis. Additional benefits include moisture retention, intrinsic antibacterial activity, biocompatibility, biodegradability, and the potential to add bioactive chemicals or nanoparticles for better healing. However, poor mechanical strength, poor stability in highly exudative settings, variability because of the degree of deacetylation, and difficulties with large-scale production and regulatory approval are some of the challenges [205]. In order to avoid these issues, chitosan is frequently mixed with synthetic polymers like PVA, PEG, and polyacrylamide or with natural polymers like gelatin, alginate, collagen, hyaluronic acid, and dextran. Microwave-aided crosslinking of chitosan-sodium alginate composite hydrogels increased swelling capacity, mechanical properties, and tissue regeneration [206]. These hydrogels accelerated re-epithelialization, increased collagen deposition, and enhanced skin architecture, indicating their potential as advanced wound dressing materials. New N-succinyl chitosan oxidized hyaluronic acid composite hydrogels prepared were found injectable and self-healing, which makes them appropriate for irregular and deep wounds [207]. Incorporation of calcium ions or PEG improved mechanical strength, biocompatibility, hemostatic activity, and wound healing, with calcium-containing hydrogels showing hemostatic efficacy comparable to commercial agents while significantly accelerating tissue repair. A ternary composite hydrogel composed of PVA, chitosan, and collagen exhibited high mechanical strength, excellent antibacterial activity, and good biocompatibility, making it effective for wound dressing applications [208]. In vivo studies demonstrated accelerated wound healing with nearly complete wound closure, enhanced epidermal regeneration, and increased hair follicle formation, indicating its strong potential for wound repair. Injectable gallic acid-modified chitosan/oxidized dextran composite hydrogels exhibited self-healing, antioxidant, antibacterial, and biocompatible properties, making them effective for treating combined radiation and burn injuries [209]. By scavenging ROS and reducing inflammation, these hydrogels significantly accelerated wound healing and tissue regeneration compared with commercial treatments. By UV photocrosslinking, methacrylate-functionalized chitosan and gelatin-based hybrid hydrogels demonstrated adjustable swelling, degradation, and interconnected porous architectures, making them appropriate for use as wound dressings [210]. These biocompatible hydrogels showed exceptional cell survival and tunable physico-chemical characteristics, underscoring their potential for wound healing and skin tissue regeneration. Table 6 presents chitosan-based formulations, highlighting their composition, functions, mechanistic outcomes, design strategy, and translational stage.

A multifunctional modular hydrogel composed of gelatin, chitosan, quercetin-loaded liposomes, and protocatechuic aldehyde–iron complexes exhibited enhanced self-healing ability, mechanical strength, antioxidant, antibacterial, and collagen-promoting properties [220]. The hydrogel effectively accelerated wound repair and showed strong potential as safe and cost-effective dressing for diabetic and drug-resistant wounds. Composite alkylated chitosan-based hydrogels and sponges have demonstrated enhanced hemostatic performance, mechanical strength, and biocompatibility. These systems improved blood absorption, clot formation, platelet activation, and coagulation, resulting in accelerated hemostasis and reduced blood loss compared with conventional wound dressings and hemostatic materials [221].

#### 4.3.2. Chitosan Derivatives-Based Hydrogels

Chitosan derivatives such as CMCS, QCS, thiolated chitosan, glycol chitosan, and N-succinyl chitosan have been developed to enhance solubility, mucoadhesion, antimicrobial activity, and overall functionality for biomedical applications. Due to its amino groups, chitosan exhibits strong mucoadhesive, antibacterial, and tissue regenerative properties, making it highly suitable for drug delivery and wound healing applications [222]. Chemical modifications and graft copolymerization further improve its solubility, biocompatibility, antioxidant activity, and therapeutic performance [223]. Graft-modified chitosan hydrogels, including chitosan-gallic acid, lactate-grafted, and N-glycosylated systems, exhibit multifunctional properties such as self-healing, adhesion, antioxidant, antimicrobial, and hemostatic activities, thereby supporting tissue repair and wound healing [224]. In addition, physical modifications through crosslinking or blending with nanoparticles, drugs, enzymes, and polymers further enhance the performance and versatility of chitosan-based composites for advanced wound care applications.

#### 4.3.3. N,N,N-Trimethyl Chitosan-Based Hydrogels

Quaternary ammonium chitosan derivatives, such as trimethyl chitosan and hydroxypropyltrimethyl ammonium chloride chitosan are synthesized through chemical modification of chitosan to improve its functionality and biomedical applicability [225]. CMCS, including N-CMCS, O-CMCS, and N,O-CMCS forms, exhibits enhanced water solubility, biocompatibility, antibacterial, moisturizing, and antioxidant properties, making it highly suitable for wound healing applications [226]. Its biological activity depends on the substitution pattern, with O-CMCS generally demonstrating stronger antibacterial activity due to the retention of amino groups [227]. A pH-sensitive and self-healing hydrogel prepared from agricultural by-products such as *Hericium erinaceus* residues and pineapple peel showed promising potential for wound healing applications [228]. In this system, aldehyde groups of oxidized microcrystalline cellulose derived from pineapple peel were crosslinked with amino groups of CMCS obtained from *Hericium erinaceus* residues through Schiff-base reaction to form the hydrogel network.

#### 4.3.4. Quaternized Chitosan (QCS)-Based Hydrogels

Dynamic QCS-based hydrogels have demonstrated strong potential for wound healing due to their self-healing, antimicrobial, and cytocompatible properties. Hydrogels formed through imine bonding between QCS and dialdehyde PEG exhibited rapid gelation, adaptability, self-healing ability, and inherent antimicrobial activity, making them suitable for irregular wound coverage and infection control [229]. Similarly, hydrogels based on N-2-hydroxypropyltrimethylammonium chloride chitosan crosslinked with β-sodium glycerophosphate formed stable thermosensitive systems with excellent cytocompatibility, enhanced cell proliferation, and increased expression of growth factors such as TGF-β1, EGF, and VEGF, thereby promoting angiogenesis and wound healing [229].

#### 4.3.5. Alkylated Chitosan-Based Hydrogels

Alkylated chitosan derivatives have been developed to overcome the low hydrophobicity and plasticity of native chitosan, resulting in improved water solubility, biocompatibility, and hemostatic properties suitable for hydrogel-based wound dressings [230]. These derivatives exhibit low hemolysis and promote platelet adhesion, aggregation, and thrombus formation, contributing to effective hemostasis without significantly affecting conventional coagulation pathways [231]. In animal studies, alkylated chitosan demonstrated effective hemostatic activity and enhanced tissue repair with minimal tissue adhesion compared to fibrin-based agents, highlighting its potential as safe wound healing material [232].

#### 4.3.6. Acetylated Chitosan-Based Hydrogels

Acetylated chitosan improves solubility, hydrophobicity, and lipophilicity by changing crystallinity, which broadens its potential applications in drug delivery and biomaterials [233]. Acetylated chitosan demonstrates excellent performance in hydrogel wound dressings due to its enhanced physicochemical properties. Various formulations, including double-network and thermoresponsive hydrogels, have been developed using acetylated or acyl-modified chitosan combined with polymers such as PVA. These hydrogels exhibit desirable features such as improved mechanical strength, flexibility, water absorption, rapid gelation, and good biocompatibility. Additionally, they support cell adhesion, proliferation, and skin regeneration, highlighting their strong potential for wound healing applications [234].

#### 4.3.7. Phosphorylated Chitosan-Based Hydrogels

Phosphorylated chitosan is a water-soluble derivative with enhanced metal-chelating, antimicrobial, hemostatic, antioxidant, anti-inflammatory, and angiogenic properties, making it highly suitable for tissue regeneration and wound healing applications [235]. This chitosan derivative-based hydrogel improves cell affinity and regulates fibroblast and endothelial cell behavior, supporting tissue regeneration and osteogenic differentiation [236]. To overcome the poor mechanical strength of native chitosan, a cellulose-inspired PVA-phosphorylated chitosan hydrogel was developed with enhanced toughness, hydration, and biocompatibility. The modified hydrogel reduced inflammatory markers and achieved approximately 95% wound healing, highlighting its potential for chronic wound care and multifunctional biomedical applications [237].

### 4.4. Hyaluronic Acid-Based Hydrogels

**Figure 7:**
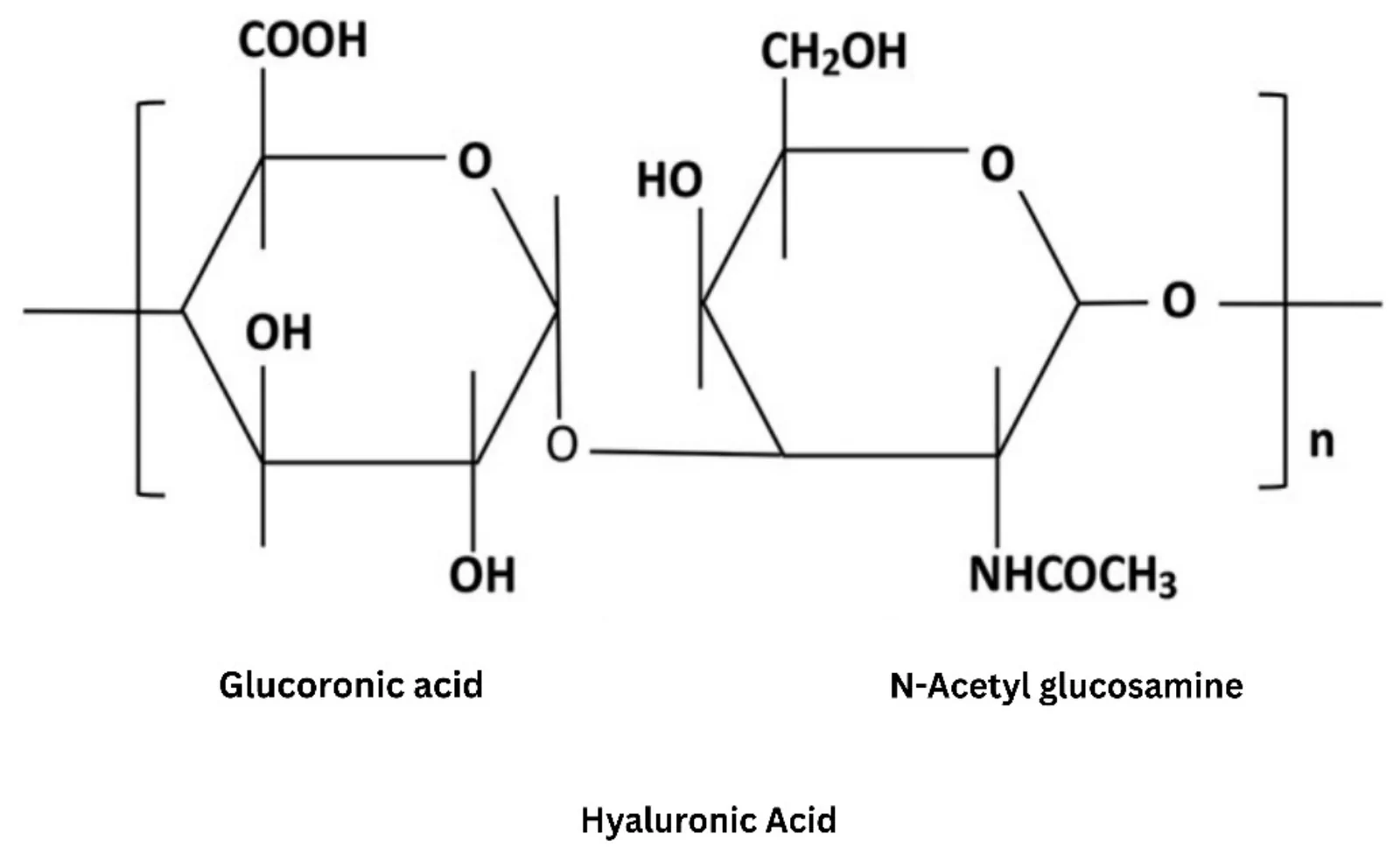
Chemical structure of hyaluronic acid.

Hyaluronic acid (HA) or Hyaluronan is a naturally occurring, linear, non-sulfated glycosaminoglycan-based biopolymer found in the ECM of the skin and other connective tissues [238]. It is composed of repeating disaccharide units of D-glucuronic acid and N-acetyl-D-glucosamine linked by alternating β-(1→3) and β-(1→4) glycosidic bonds as shown in Figure 7. HA is generally classified according to its molecular weight, such as extremely high (>6000 kDa), high (>1000 kDa), low (10–1000 kDa), and oligosaccharides (<10 kDa) [239]. Indeed, its biological activity is largely determined by the molecular weight, and various size ranges usually result in diverse or even opposite effects. This glycosaminoglycan is highly hydrophilic due to the presence of carboxyl and hydroxyl groups, enabling it to retain up to 1000 times its weight in water and thereby contributing to tissue hydration, viscoelasticity, and matrix organization [240]. Its structure is stabilized by hydrogen bonding, forming helices that further aggregate into networks through intermolecular interactions. Network strength and viscosity increase with molecular weight and concentration, giving it non-Newtonian, shear-thinning behavior. Its rheological properties depend on pH, temperature, and ionic strength, while extreme pH conditions (<4 or >11) cause reversible degradation of the polymer network [241]. This gel-to-liquid transition is reversible. These physicochemical properties are essential for its biological functions and its wide range of biomedical applications. Hyaluronan plays crucial role in wound healing as both structural and signaling molecule. High molecular weight hyaluronic acid maintains hydrated ECM, supports tissue integrity, and facilitates cell migration, while also interacting with cellular receptors to regulate inflammation, proliferation, and tissue repair [242].

At the molecular level, HA promotes wound healing by interacting with cell surface receptors that regulate inflammation, cell migration, and tissue regeneration. HA interacts with cluster of differentiation 44 (CD44) and receptor for hyaluronan-mediated motility (RHAMM) receptors to regulate keratinocyte migration, angiogenesis, and ECM remodeling while suppressing excessive inflammation and ROS, particularly in diabetic wounds [243].

The anti-inflammatory activity of HA is closely associated with its molecular weight and interactions with cell-surface receptors. High-molecular-weight HA binds primarily to CD44 receptors on immune and stromal cells, suppressing NF-κB-mediated inflammatory signaling, reducing leukocyte infiltration, and decreasing the release of pro-inflammatory cytokines, including TNF-α, IL-1β, and IL-6. HA also regulates macrophage polarization toward the M2 phenotype, promotes fibroblast migration and proliferation, enhances angiogenesis, and supports extracellular matrix remodeling, thereby accelerating tissue regeneration. In contrast, low-molecular-weight HA fragments generated during tissue injury may transiently activate inflammatory pathways through Toll-like receptors (TLR2 and TLR4), highlighting the importance of HA molecular weight in determining its biological activity [239,244].

The incorporation of diverse bioactive components including stem cells, growth factors, and peptides, offers versatile and synergistic approaches for disease treatment, particularly in areas such as wound healing and regenerative medicine. Stem cells promote healing mainly through paracrine signaling by releasing bioactive molecules known as stem cell-derived conditioned media. Lyophilized conditioned media offer advantages over direct stem cell therapy, including easier storage, lower immunogenicity, and absence of tumor risk [245]. Amnion-derived conditioned media incorporated into modified hyaluronic acid hydrogels demonstrated sustained release, improved mechanical properties, enhanced angiogenesis, and immune regulation, leading to accelerated diabetic wound healing [246]. Growth factors that promote angiogenesis, cell proliferation, and collagen synthesis like EGF, VEGF, and platelet-derived growth factor are vital to wound healing. EGF-loaded hyaluronic acid–collagen scaffolds that are localized and prolonged therapeutic effects have shown antioxidant effect, enhanced diabetic wound healing while decrease in inflammation [247]. Similarly, ECM-derived peptides also support wound healing by imitating natural biological signals that control cell migration, proliferation, and differentiation. Peptides formed from fibronectin, laminin, and collagen promote cell adhesion, angiogenesis, and the function of fibroblasts and keratinocytes, promoting tissue regeneration. For instance, hydrogels modified with the laminin-derived pentapeptide IKVAV substantially increased cell proliferation, migration, and gene expression, exhibiting high regenerative capacity [248].

Antimicrobial peptides have received a lot of interest in wound healing because of their broad-spectrum antibacterial effect, minimal resistance potential, and capacity to promote immunological responses and tissue regeneration [249]. However, their clinical use is limited due to instability and quick degradation in wound settings. In order to solve these issues, small peptide functionalized HA hydrogels were designed to enhance stability, antibacterial activity, and wound healing efficiency. Acrylated hyaluronic acid hydrogels conjugated with the antimicrobial peptide PP4-3.1 demonstrated rapid gelation, stable mechanical properties, enhanced cell adhesion, and strong antibacterial activity against *S. aureus* and *E. coli* [250]. Similarly, peptide-saccharide composite hydrogels combining hyaluronic acid with modified diphenylalanine exhibited good biocompatibility, self-healing ability, sustained drug release, and enhanced diabetic wound healing [251]. Injectable pH-responsive hyaluronan-based hydrogels crosslinked with antimicrobial peptides also showed potent antibacterial activity and effectively promoted healing of chronically infected wounds. In addition, peptide-modified 3D printable hyaluronic acid hydrogels loaded with bioactive peptides improved fibroblast viability and swelling behavior, highlighting their potential for advanced wound healing applications [252].

Phytochemicals incorporated into HA-based hydrogels play significant role in enhancing wound healing by providing antioxidant, anti-inflammatory, and antimicrobial effects while promoting tissue regeneration. The rise of antibiotic-resistant wound infections highlights the need for advanced wound healing materials incorporating bioactive phytochemicals. A HA-curcumin hydrogel was developed to improve curcumin stability and activity [253]. It showed strong antibacterial effects against *P. aeruginosa*, including inhibition of biofilm formation and quorum sensing, and promoted rapid, scar-free wound healing in vivo. Tannins such as ellagic acid promote wound healing by exerting antioxidant and anti-inflammatory effects, enhancing collagen synthesis, and accelerating tissue regeneration and epithelialization [254]. Diabetic wounds are difficult to treat due to prolonged inflammation, reduced angiogenesis, and impaired ECM remodeling. To address these challenges, collagen-HA scaffold loaded with epigallocatechin gallate was developed [255]. The scaffold demonstrated porous architecture, sustained drug release for up to 14 days, and good biocompatibility. In diabetic rat model, it significantly accelerated wound closure compared to controls. Additionally, it promoted epidermal and dermal regeneration, enhanced fibroblast proliferation and angiogenesis, reduced inflammation, and improved collagen deposition and tensile strength. Moreover, various phytochemicals have been widely utilized in wound healing, including alkaloids (berberine), saponins (ginsenosides Rg1), terpenoids (thymol), phenolic compounds (resveratrol), and polysaccharides (acemannan). These compounds collectively contribute to wound healing through antimicrobial, anti-inflammatory, antioxidant, and tissue regenerative mechanisms [256].

Similarly, hyaluronic acid was also used in formulating various synthetic therapeutics aimed for wound healing. Gas molecules have emerged as effective and safe therapeutic agents in tissue repair, gaining significant attention in recent years. Gas therapy contributes to all stages of wound healing by modulating inflammation and infection, promoting cell proliferation and migration, enhancing angiogenesis, and supporting ECM remodeling [257]. Key therapeutic gases include oxygen, nitric oxide, carbon monoxide, hydrogen sulfide, and others such as sulfur dioxide and hydrogen. Advances in this field have led to the development of gas-releasing systems, including gas-producing and gas-loaded materials, enabling controlled and sustained delivery for improved wound healing outcomes. For example, HA-based gas-entrapping materials loaded with carbon monoxide have been developed to enhance wound healing [258]. Such systems provide constant gas release for 48 h after a brief burst release. When compared to traditional dressings, the new formulation significantly expedited wound closure in porcine full-thickness wound model, shortening the healing period by almost 14 days. Their capacity as effective topical treatments for tissue repair were demonstrated by this improvement, which was related to increased neovascularization, decreased inflammation, and increased epithelialization.

Despite hyaluronan-based hydrogels show promise as wound dressings, their efficacy in diabetic wounds is limited due to bacterial infection, high oxidative stress, extended inflammation, and decreased nitric oxide levels. This resulted in the development of an injectable, self-healing HA hydrogel incorporating allomelanin-BNN6 nanoparticles [259]. Effective ROS scavenging and regulated nitric oxide release under near-infrared (NIR) irradiation are shown by this nanocomposite system with minor photothermal antibacterial effects. It significantly reduces oxidative stress, controls infection, promotes angiogenesis, and enhances vascular regeneration, thereby accelerating diabetic wound healing. To further enhance wound healing, the combined therapeutic effects of HA and nitric oxide were integrated into single system by chemically modifying hyaluronic acid via carbodiimide-mediated coupling [260]. The resulting HA derivatives exhibited controlled nitric oxide release with tunable kinetics under physiological conditions. Notably, the 6 kDa HA variant demonstrated potent antibacterial activity against resistant wound pathogens, including MRSA and *P. aeruginosa*, in both planktonic and biofilm forms. In infected wound models, the nitric oxide-releasing HA accelerated wound closure and reduced bacterial load while maintaining biodegradability through natural enzymatic pathways. HA-based hydrogels have also been developed for localized antimicrobial therapy. Gentamicin-crosslinked HA hydrogels enabled sustained antibiotic release for up to 9 days while allowing adjustable adhesive and cohesive properties, thereby improving infection control in wounds [261]. In addition, collagen-HA composite dressings loaded with silver nanoparticles or gentamicin demonstrated broad-spectrum antimicrobial activity and enhanced wound healing, with formulations containing either silver nanoparticles or gentamicin individually showing superior performance compared to combined formulations [262].

#### 4.4.1. Hyaluronic Acid-Based Multifunctional Hydrogels

Multifunctional HA-based hydrogels have emerged as advanced wound dressing platforms due to their ability to respond to external stimuli and provide controlled therapeutic effects. These hydrogels can be engineered to exhibit photo-responsive, glucose-responsive, electro-responsive, self-healing, adhesive, antioxidant, and antibacterial properties, thereby enhancing wound healing efficiency and tissue regeneration. Stimuli-responsive HA hydrogels enable controlled drug release in response to environmental changes such as light, glucose concentration, pH, and electrical stimulation [263,264]. Furthermore, incorporation of conductive polymers, silver nanoparticles, plant-derived compounds, and antimicrobial functionalities significantly improves antibacterial performance, angiogenesis, cell migration, and inflammation control. Collectively, these multifunctional systems represent promising next-generation wound dressings for chronic, diabetic, and antibiotic-resistant wound management [265,266,267]. In this context, a study developed HA-based hydrogel containing EGF and keratinocyte growth factor to improve corneal wound healing through controlled growth factor release [268]. EGF promoted epithelial regeneration, while the latter enhanced stromal repair, and their combined action supported efficient corneal regeneration and improved wound healing outcomes. A multifunctional chitosan/HA hydrogel loaded with quercetin was developed for NIR-triggered nitric oxide release, enabling synergistic antibacterial and wound healing effects [269]. It exhibited good injectability, self-healing ability, adhesion, antioxidant activity, and biocompatibility. The quercetin–Fe^3+^ complex provided photothermal activity for controlled nitric oxide release and sustained antibacterial action even without NIR irradiation. Magnetic nanocarriers with iron-modified montmorillonite core coated with chitosan/ HA shell and loaded with curcumin and ciprofloxacin were also developed [270]. The prepared nanocarriers exhibited efficient drug loading and sustained, pH-responsive release, which was further enhanced under external magnetic field. Additionally, scratch assay results confirmed their potential application in wound healing. Table 7 highlights multifunctional and stimuli-responsive HA-based hydrogels developed for wound healing.

#### 4.4.2. Hyaluronic Acid-Based Composite Hydrogels

Self-healing composite hydrogels are highly promising for wound healing because they can restore their structural integrity and functionality after damage through reversible physical interactions and dynamic chemical bonds, closely mimicking the ECM [271]. Their self-repair capability supports cell adhesion, migration, proliferation, and tissue regeneration, making them suitable for advanced wound dressings and regenerative therapies [272]. These hydrogels can heal spontaneously or in response to stimuli like pH, temperature, light, or glucose levels. This is especially beneficial in environments involving chronic wounds. Injectable and in situ-forming hydrogels that effectively adapt to wound sites and replicate natural skin are examples of recent advancements.

Recent advances in HA-based composite hydrogels have focused on biomimetic ECM design, injectable systems, and dual-crosslinking strategies to enhance wound healing performance. Collectively, these approaches improve mechanical strength, self-healing capability, swelling behavior, chemical stability, controlled degradation, and sustained therapeutic release while maintaining excellent cytocompatibility [273,274,275,276,277]. Functional modifications, including incorporation of collagen, ferrocene, and dual-crosslinking chemistries, further promote fibroblast and endothelial cell proliferation, angiogenesis, collagen deposition, exudate management, infection control, and accelerated tissue regeneration, making these hydrogels promising platforms for chronic wound management and drug delivery [273,274,275,276,277]. Despite these advantages, challenges remain in optimizing the balance between mechanical strength and biodegradability, ensuring long-term in vivo stability, achieving scalable manufacturing, and generating sufficient clinical evidence to support widespread clinical translation. HA-based formulations incorporating diverse active ingredients and their functional contributions to wound healing are presented in Table 8.

### 4.5. Sodium Alginate-Based Hydrogels

**Figure 8:**
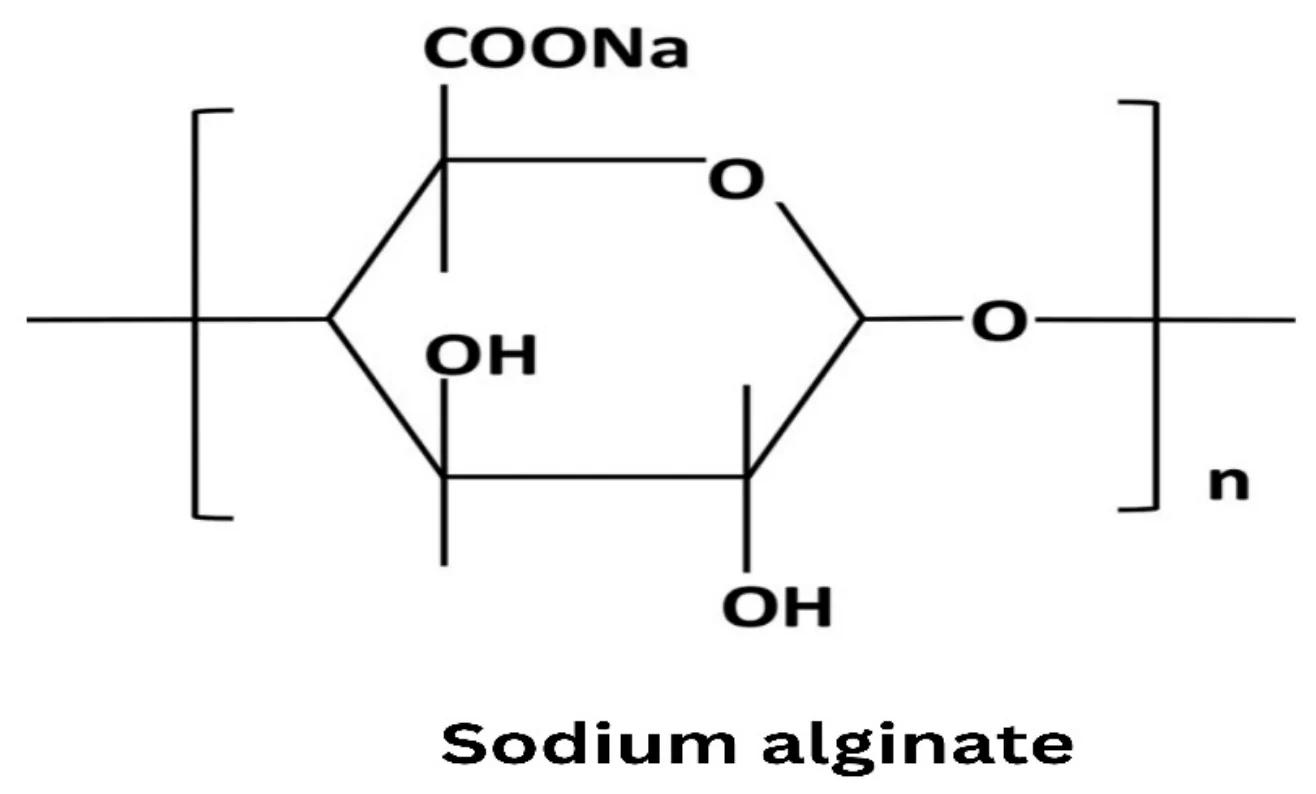
Chemical structure of sodium alginate showing the repeating β-D-mannuronic acid (M) and α-L-guluronic acid (G) units.

Sodium alginate (SA) is a natural anionic polysaccharide derived from brown algae, consisting of β-D-mannuronic and α-L-guluronic acid units linked by 1,4-glycosidic bonds, forming block copolymer as shown in Figure 8. The network structure of SA hydrogels consists of three-dimensional network formed through the physical or chemical crosslinking of polymer chains [285]. Physical crosslinking in SA hydrogels is commonly achieved through freeze–thaw cycles or ionic interactions with multivalent cations such as Ca^2+^. These cations interact electrostatically with the carboxyl and guluronic acid groups of alginates to form ionic egg-box structures, producing stable and reversible three-dimensional hydrogel network with tunable mechanical and swelling properties [286].

Physical entanglement in SA hydrogels involves the interwinding of polymer chains without chemical bonds, forming three-dimensional network that enhances viscoelasticity, mechanical strength, and shear resistance. On the other hand, chemical crosslinking typically refers to the use of a crosslinking agent and involves the formation of covalent bonds. These hydrogels are excellent for biomedical applications, especially as wound dressings, given their high-water content and soft tissue-like structure. Photopolymerization is rapid and controllable chemical crosslinking method for SA hydrogels, where light-activated photoinitiators generate free radicals that initiate polymerization, enabling uniform, in situ network formation with tunable mechanical and functional properties [287]. Schiff base crosslinking in SA hydrogels involves reactions between carbonyl and amine groups to form dynamic imine bonds, enabling the formation of stable, biocompatible, and self-healing hydrogels with enhanced mechanical and functional properties. Click chemistry enables efficient and selective covalent crosslinking in SA hydrogels under mild conditions, forming stable networks with enhanced mechanical strength, biocompatibility, and tunable degradability [288]. Polycondensation crosslinking forms stable three-dimensional alginate networks via reactions with crosslinking agents, with careful consideration of biocompatibility. Oxime bond crosslinking forms biocompatible, reversible, and stimuli-responsive alginate hydrogels with tunable properties, requiring characterization to assess their structure and performance [289]. At the molecular level, alginate promotes wound healing by modulating the wound microenvironment and supporting key cellular processes involved in tissue repair. Alginate primarily supports wound repair by maintaining a moist microenvironment, modulating inflammatory cytokines, and enhancing fibroblast proliferation and ECM deposition through sustained release of therapeutic agents [290]. SA-based hydrogels are widely explored for wound healing because they stimulate cytokine production, such as IL-6 and TNF-α, thereby supporting tissue repair [27].

Phytochemicals are increasingly incorporated into SA hydrogels to enhance wound healing due to their natural bioactivity and biocompatibility [291]. These phytochemicals when incorporated into alginate hydrogels, produce controlled drug release, which can promote angiogenesis, tissue regeneration, and cell proliferation. This combination increases alginate-based dressings’ therapeutic efficacy, making them ideal options for advanced wound treatment. In this regard, curcumin-loaded alginate hydrogels have shown strong biodegradability, prolonged curcumin release, and an interconnected porous structure that is beneficial for cell infiltration and nutrient exchange [292]. Remarkable biocompatibility and fibroblast proliferation were shown by the hydrogel, specifically at suitable curcumin dose of 0.1 mg/mL. In vivo studies demonstrated accelerated wound closure (>80% within 14 days) and downregulation of MMP-9, indicating reduced inflammation and extracellular matrix degradation. Additionally, the expression of key wound-healing mediators, including TGF-β1 and VEGF, was upregulated, thereby promoting tissue regeneration and angiogenesis. The efficacy of SA/chitosan hydrogels containing crocetin at various levels (0, 25, 75, 150, and 300 µM) for wound healing was investigated [293]. Administration of prepared hydrogel loaded with crocetin (150 µM) significantly enhanced healing progress in comparison to lower and higher concentrations, as demonstrated by the in vivo evaluation using full-thickness wound models. This combination enhanced tissue remodeling, accelerated wound contraction, and facilitated re-epithelialization. In addition, molecular analysis revealed elevation of key healing-related markers, such as VEGF, TGF-β, and collagen types I and III, indicating increased angiogenesis and ECM deposition. To overcome the mechanical weakness and brittleness of SA, natural fibers such as kapok and hemp were incorporated to enhance strength while retaining flexibility for wound dressing applications [294]. Both fibers improved strength, with hemp showing superior performance (185 cN, 1.48% elongation) compared to kapok (174 cN,1.24% elongation), while maintaining high exudate absorbency (~432–435%).

**Figure 9:**
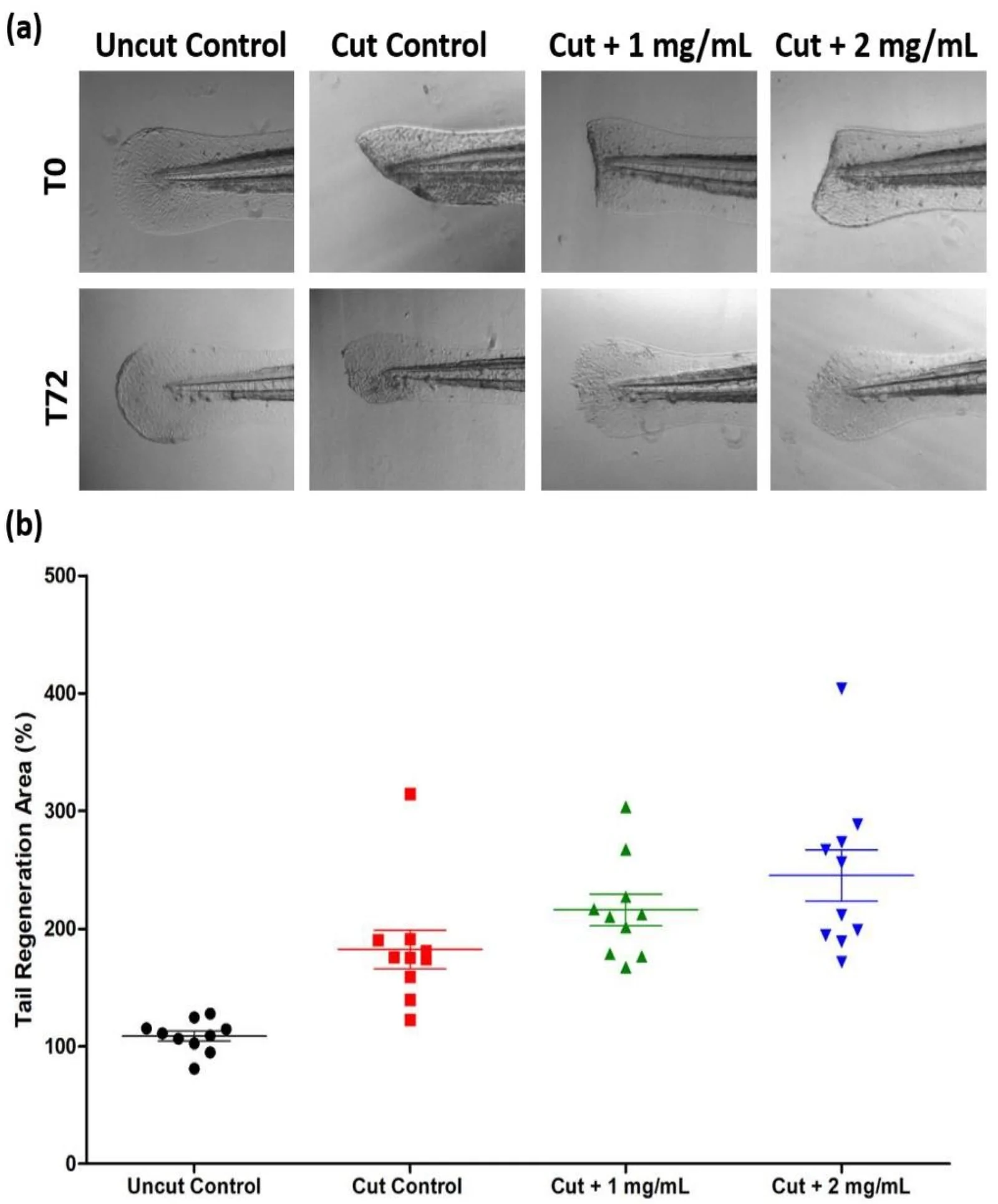
In vivo wound healing evaluation in zebrafish embryos following treatment with moringa oleifera leaf extract/sodium alginate/polyvinyl alcohol scaffold suspension (S5). Representative images of caudal tail fin regeneration were captured from 0 to 72 h post-treatment in untreated and treated groups with 1 and 2 mg/mL scaffold concentrations (**a**). Quantitative analysis of tail regeneration length demonstrated enhanced regenerative capacity of the S5 scaffold compared to the control group (**b**). Reproduced from Ref. [295].

Hydrogel-based SA/PVA scaffolds were successfully fabricated using the freeze-thawing technique for wound healing applications [295]. Moringa oleifera leaf extract, particularly the 80% methanolic extract with the highest antioxidant activity, was incorporated into the SA/PVA hydrogel scaffolds at different concentrations (0.0–2.5%). After 3 days, the extract-loaded hydrogel scaffolds significantly enhanced in vivo wound healing, increasing tissue growth from 216 ± 42% (1 mg/mL) to 246 ± 69% (2 mg/mL), compared to 182 ± 51% in transected embryos and 109 ± 14% in uncut embryos (Figure 9). Raphanus sativus-loaded SA hydrogels demonstrated promising wound healing and antimicrobial potential. In vivo studies confirmed enhanced healing activity compared to the control, highlighting its potential application in cutaneous wound repair [296]. In a recent study, green-synthesized Ag/Fe_3_O_4_ bimetallic nanoparticles using *Mimusops elengi* leaf extract were incorporated into SA hydrogels, resulting in enhanced antioxidant, antimicrobial, and wound healing activities, with complete wound recovery observed within 21 days [297].

SA hydrogels containing active agents such as antimicrobials, anti-inflammatory drugs, nanoparticles, and growth-promoting compounds have shown considerable potential in advanced wound care. Incorporation of silver nanoparticles into alginate-based hydrogels further enhanced antibacterial activity, mechanical strength, water absorption, and biocompatibility, making them effective for infection control and moist wound management [298]. In addition, SA hydrogels exhibit intrinsic hemostatic properties, supporting platelet aggregation and fluid absorption, which are beneficial for highly exuding wounds such as burns and diabetic ulcers [299]. The incorporation of graphene oxide into alginate-based hemostatic powders further improved coagulation efficiency, water absorption, and wound healing through rapid gel formation, Ca^2+^ release, and ROS scavenging [300]. Despite persistent issues with long-term mechanical stability and functionality, SA hydrogels remain a unique and promising platform for next-generation wound care applications. Recent research trends have focused on producing advanced alginate-based composite hydrogels, particularly stimuli-responsive, antimicrobial, and bioactive systems, to overcome limitations such as inadequate mechanical strength and quick degradation. Innovative ideas such as double-network hydrogels, nano-reinforced systems, and smart hydrogels are being investigated in order to increase performance and provide multifunctional wound healing systems [301].

SA hydrogels can also play a crucial role in the biological stages of wound healing, which include inflammation, proliferation, and remodeling. Another important attribute of sodium alginate hydrogels is their potential to hold and release growth factors steadily. This targeted delivery technique increases treatment efficacy while reducing systemic side effects. Hybrid SA-based hydrogel systems showed significant potential for wound healing and tissue regeneration. A PVA/alginate hydrogel with bFGF-loaded polycaprolactone microspheres provided constant growth factor release, high biocompatibility, and improved tissue regeneration, facilitating burn wound healing despite minor decrease in mechanical strength [302]. Moreover, 3D alginate microsphere-collagen hydrogel loaded with human umbilical cord mesenchymal stem cells led to prolonged cell release, greater collagen deposition, better re-epithelialization, and almost complete wound healing by day 21. The system also increased angiogenic factors like VEGF and FGF2, showing its efficacy as an improved regenerative wound healing platform [303]. SA-based wound dressings showed considerable therapeutic potential by incorporating bioactive substances and stem cells. A SA/hydrogen sulfide donor (JK-1) hydrogel dressing enabled pH-responsive and sustained gas release, maintaining moist wound environment while enhancing granulation tissue formation, collagen deposition, angiogenesis, and re-epithelialization, thereby accelerating wound healing [304]. Similarly, SA-platelet-rich plasma hydrogels provided sustained release of growth factors such as EGF and VEGF, promoting cell proliferation, angiogenesis, and improved wound closure [305]. In diabetic wounds, alginate/gelatin hydrogels loaded with adipose-derived mesenchymal stem cells enhanced healing by stimulating angiogenesis, collagen deposition, granulation tissue formation, and wound closure, while also modulating inflammation through reduced pro-inflammatory cytokines and increased anti-inflammatory cytokines and M2 macrophage polarization [306].

#### Sodium Alginate-Based Composite Hydrogels

SA hydrogels are extensively used in wound dressings because of their excellent absorbency, moisture retention, biocompatibility, and gel-forming properties, which produce optimal environment for wound healing. These hydrogels absorb wound exudate, reduce infection risk, and promote granulation tissue formation, collagen synthesis, tissue repair, and re-epithelialization. Their hemostatic nature also supports platelet aggregation and coagulation, accelerating the early stages of wound healing [307]. In addition, this hydrogel can closely conform to wound surfaces, minimizing dead space and enhancing protection against microbial contamination. Recent advancements in SA-based wound dressings focus on multifunctional composite hydrogels with enhanced antibacterial, antibiofilm, antioxidant, and regenerative properties. Incorporation of nanomaterials such as Ti_3_C_2_Tx MXene, Zn^2+^, β-Ga_2_O_3_ nanoparticles, and QCS has significantly improved antibacterial efficacy through photothermal and chemical mechanisms, while also promoting cell migration and proliferation. Alginate–chitosan composite hydrogels have further demonstrated excellent drug delivery capabilities and tissue regeneration potential. For example, EGF-loaded N-CMCS-alginate hydrogels enhanced cell proliferation and wound repair, while zinc-doped bioactive glass incorporated into alginate/chitosan systems improved antibacterial activity and maintained moist healing environment [308]. Advanced formulations such as SA-chitosan oligosaccharide–zinc oxide hydrogels exhibited high porosity, swelling capacity, water vapor permeability, strong antibacterial activity, and excellent biocompatibility. In vivo studies demonstrated significantly accelerated burn wound healing, enhanced collagen deposition, fibroblast proliferation, reduced inflammation, and regeneration of skin appendages compared to conventional treatments [309]. Furthermore, newly developed multifunctional SA hydrogels fabricated through photo-crosslinking or Schiff base interactions displayed desirable mechanical strength, tissue adhesion, self-healing ability, and hemostatic activity, while also promoting neovascularization and wound repair in burn models [307]. Despite challenges related to mechanical stability and degradation, sodium alginate-based hydrogels remain promising candidates for next-generation multifunctional wound dressings. Sodium alginate-based formulations incorporating diverse bioactive agents and their functional contributions to wound healing are summarized in Table 9.

Each natural biopolymer possesses distinct advantages and limitations that influence its suitability for specific wound-healing applications. Cellulose exhibits excellent mechanical strength and moisture retention but has limited intrinsic bioactivity. Chitosan possesses inherent antibacterial and hemostatic properties but generally requires reinforcement to improve its mechanical stability. However, its antibacterial activity is highly pH-dependent, with maximal activity under acidic conditions (typically pH < 6.5) due to protonation of its amino groups, whereas it is considerably reduced at physiological pH. Alginate demonstrates superior swelling capacity and exudate absorption, making it particularly suitable for highly exuding wounds, although its mechanical strength is relatively low without crosslinking. Hyaluronic acid closely mimics the native extracellular matrix and promotes cell migration and tissue regeneration but undergoes rapid enzymatic degradation and therefore often requires chemical modification or crosslinking to enhance its stability. These complementary characteristics have driven the development of composite and hybrid hydrogels that combine the advantages of multiple biopolymers while minimizing their individual limitations. To facilitate comparison among the major natural biopolymers used in wound healing, Table 10 summarizes their key physicochemical characteristics, mechanical performance, swelling behavior, degradation mechanisms, antibacterial activity, drug delivery capability, preferred wound applications, major limitations, and clinical applicability. Furthermore, representative in vivo studies have been comparatively summarized in Table 11 according to wound model, animal species, treatment duration, and major wound healing outcomes. Table 12 provides a comparative overview of representative preclinical biopolymer-based hydrogel systems based on their drug loading/release characteristics, antimicrobial activity, and wound healing efficacy. Current literature indicates that chitosan-based hydrogels are widely investigated for infected wounds owing to their intrinsic antibacterial and hemostatic properties, whereas alginate-based systems are particularly suitable for highly exuding wounds due to their excellent fluid absorption and gel-forming capacity [27,310]. HA-based hydrogels show strong potential for chronic and regenerative wound healing because of their extracellular matrix-mimicking properties and ability to promote cell migration, angiogenesis, and tissue remodeling [311]. Cellulose-based hydrogels, especially bacterial cellulose and cellulose derivatives, are commonly explored for moisture retention, mechanical reinforcement, and sustained drug delivery [312]. Therefore, the selection of a suitable biopolymer should be guided by wound type, exudate level, infection status, desired mechanical properties, and therapeutic delivery requirements.

**Table 9:** Sodium alginate-based wound healing formulations and their composition, functions, mechanistic outcomes, design strategy, and translational stage.

Composition	Wound Healing Functions	Mechanistic/Functional Outcomes	Wound Type	Hydrogel Design Strategy	Translational Stage	References
Naringenin	Promotes wound closure, tissue regeneration, antibacterial activity	Porous and biodegradable hydrogel supports moist environment, sustained release of naringenin, improves cell viability (cytocompatible), enhances in vivo wound closure (especially at higher concentration, 20%).	Full-thickness excisional wound	Bioactive flavonoid-loaded hydrogel	In vitro + In vivo	[313]
Quercetin	Antioxidant, anti-inflammatory, supports diabetic wound healing	Reduces oxidative stress and inflammation, provides moist, antimicrobial environment via chitosan/alginate hydrogel, promotes wound contraction, supports cell adhesion and biocompatibility.	Diabetic wound	Bioactive-loaded composite hydrogel	In vitro + In vivo	[314]
*Capparis spinosa* L. extract	Antioxidant, antibacterial, moisture retention, promotes cell proliferation, structural scaffold	High-swelling porous hydrogel enhanced water retention, antioxidant and antibacterial activity against *E. coli* and *S. aureus*, and promoted cell proliferation and wound healing.	Full-thickness wound	Plant extract-loaded hydrogel	In vitro	[315]
Amikacin	Antibacterial, moisture retention, controlled drug delivery, structural support, promotes re-epithelialization and granulation	Thermosensitive porous hydrogel enabled sustained amikacin release, enhanced antibacterial activity against *S. aureus* and *P. aeruginosa*, and promoted wound closure and tissue regeneration.	Infected wound	Thermosensitive drug-loaded hydrogel	In vitro + In vivo	[316]
Betula utilis bark extract	Antibacterial, antioxidant, moisture retention, promotes wound contraction and re-epithelialization	Exhibited good mechanical strength, potent antibacterial and antioxidant (>90%) activity, and promoted (~93%) wound contraction with complete re-epithelialization.	Excisional wound	Plant extract-loaded hydrogel	In vitro + In vivo	[317]
Chlorogenic acid	Antibacterial, moisture regulation, controlled drug release, promotes tissue regeneration and repair	Enhances antibacterial activity, maintains optimal wound moisture, provides mechanical stability and biocompatibility, enables sustained drug release, and promotes >90% wound healing with improved tissue regeneration.	Full-thickness wound	Controlled drug delivery composite hydrogel	In vitro	[318]
Benlysta (belimumab)	Promotes cell proliferation, anti-inflammatory, controlled drug delivery, supports tissue regeneration	Enhanced proliferation of HaCaT and L929 cells, reduced macrophage-mediated inflammation, and provided controlled drug release to promote tissue repair.	Chronic wound	Injectable immunomodulatory hydrogel	In vitro + In vivo	[319]

**Table 10:** Comparative overview of the major natural biopolymers used in hydrogel wound dressings, highlighting their physicochemical characteristics, mechanical performance, swelling behavior, degradation mechanisms, antibacterial activity, drug delivery capability, wound applications, limitations, and clinical applicability.

Parameter	Cellulose and Derivatives	Chitosan and Derivatives	Alginate	Hyaluronic Acid
**Source**	Plant cell walls, bacterial cellulose	Chitin (crustacean shells, fungi)	Brown seaweed	Extracellular matrix of connective tissues
Physicochemical characteristics	Hydrophilic, porous, high-water absorption, excellent film-forming ability	Cationic, hydrophilic, mucoadhesive, film-forming	Anionic, highly hydrophilic, ionically crosslinkable	Highly hydrophilic, viscoelastic, ECM-mimetic
Mechanical performance	Good mechanical strength; enhanced by crosslinking/composites	Moderate; often reinforced through blending or crosslinking	Moderate to low; improved by composite formation	Moderate; requires chemical modification or crosslinking for stability
Swelling capacity	High water-holding capacity; excellent moisture retention	High; promotes moist wound environment	Very high; excellent exudate absorption	High; retains moisture and maintains hydrated wound bed
Biodegradability	Biodegradable (derivative-dependent)	Excellent	Excellent	Excellent
Degradation mechanism	Slow hydrolytic/enzymatic degradation (derivative-dependent)	Enzymatic degradation by lysozyme	Ionic exchange and hydrolytic degradation	Enzymatic degradation by hyaluronidase
Biocompatibility	Excellent	Excellent	Excellent	Excellent
Intrinsic antibacterial activity	Limited (except when functionalized)	Moderate (pH-dependent)	Limited	Limited
Drug delivery capability	Excellent; suitable for sustained and stimuli-responsive delivery	Excellent; suitable for proteins, peptides, and small molecules	Excellent; controlled release through ionic gelation	Excellent; suitable for growth factors, proteins, stem cell-derived products, and biologics
Common modification strategies	Chemical derivatization, nanocellulose reinforcement, composite hydrogels	Quaternization, carboxymethylation, nanoparticle incorporation, composite hydrogels	Covalent/ionic crosslinking, nanoparticle incorporation, double-network hydrogels	Methacrylation, aldehyde modification, dual crosslinking, composite hydrogels
Preferred wound applications	Burns, chronic wounds, skin substitutes	Infected wounds, diabetic ulcers, hemostatic dressings	Highly exuding wounds, burns, pressure ulcers	Chronic wounds, diabetic ulcers, regenerative wound healing
Major limitations	Limited intrinsic bioactivity; requires functionalization	Moderate mechanical strength; rapid degradation in acidic environments	Poor mechanical stability without crosslinking	Rapid degradation and limited mechanical strength
Clinical applicability	Commercial dressings and active research	Commercial dressings and advanced wound therapeutics	Widely used clinically for exudative wounds	Clinically used and extensively investigated in regenerative medicine

**Table 11:** Comparative summary of representative preclinical studies on biopolymer-based hydrogel wound dressings.

Biopolymer	Wound Model	Animal Species	Treatment Period	Main Healing Outcomes	Representative Reference
Carboxymethyl chitosan/CMC	Full-thickness diabetic wound	Streptozotocin-induced diabetic rat	21 days	Enhanced re-epithelialization, increased collagen deposition and angiogenesis, reduced inflammation, and improved diabetic wound healing	[100]
Hydroxyethyl cellulose/HEC	Full-thickness thermal burn wound	Male Wistar rat	5 days	Increased granulation tissue formation, increased angiogenesis and re-epithelialization, higher Ki-67-positive cell proliferation, and improved tissue regeneration compared with control groups	[141]
Hydroxypropyl methyl cellulose/HPMC	Full-thickness excisional wound	Wistar rat	12 days	Enhanced wound contraction (≈50% wound healing achieved earlier than the positive control), reduced inflammation (decreased MPO activity), enhanced angiogenesis, improved epidermal regeneration, and overall wound healing comparable to the commercial dressing (Dersani^®^).	[176]
Chitosan	Streptozotocin (STZ)-induced diabetic full-thickness skin wound	Diabetic Sprague–Dawley rat	14 days	Enhanced angiogenesis (↑CD31 and VEGF expression), increased collagen deposition, improved re-epithelialization, reduced inflammation, and superior tissue regeneration compared with control groups.	[211]
Hyaluronic acid/HA	Streptozotocin (STZ)-induced diabetic full-thickness skin wound	Diabetic Sprague–Dawley rat	21 days	Enhanced angiogenesis, increased collagen deposition, improved re-epithelialization, and promoted regeneration of skin appendages through glucose-responsive antioxidant activity.	[266]
Alginate	Full-thickness excisional wound	Wistar rat	14 days	Increased collagen deposition and re-epithelialization, reduced inflammation, increased fibroblast proliferation and granulation tissue formation, with improved overall wound healing compared with untreated and blank hydrogel groups.	[313]

**Table 12:** Comparison of representative biopolymer-based hydrogel systems in the preclinical stage of development based on drug loading/release characteristics, antimicrobial activity, and wound healing efficacy.

Hydrogel System	Drug Loading/Release	Antimicrobial Activity	Wound Closure/Healing Outcome	Ref.
CMC-based curcumin hydrogel	pH-responsive controlled release	Antibacterial	Accelerated full-thickness wound closure; reduced inflammation and scarring	[107]
CMC/ZnO nanocomposite hydrogel	Not reported	>70% antibacterial activity against *S. aureus* and *E. coli*	Effective wound healing within 24 h	[111]
HEC/PVA-curcumin hydrogel film	Curcumin-loaded	Antibacterial	~95% wound closure within 12 days	[128]
HEC peptide hydrogel	LysSYL or dual-drug loading	Potent anti-MRSA activity	95.1% wound healing within 14 days; 86.1% closure within 9 days	[131]
HPMC hydrogel with ofloxacin/suture fibers	Controlled ofloxacin release	Broad-spectrum antibacterial activity	~95% wound closure in 14 days; increased collagen deposition	[171]
Chitosan/palmitoyl tetrapeptide-7 hydrogel with stem cells	Stem cell-loaded	Antibacterial	>95% wound closure; reduced TNF-α/IL-6; enhanced angiogenesis and collagen deposition	[211]
Alginate hydrogel with *Capparis spinosa* extract	Plant extract-loaded	Active against *E. coli* and *S. aureus*	Improved cell viability, proliferation, and wound healing	[315]
Alginate hydrogel with Betula utilis extract	Extract incorporated	Significant antibacterial activity	~93% wound contraction and complete re-epithelialization	[317]
HA-based glucose-responsive antioxidant hydrogel	Glucose-responsive therapeutic release	Indirect infection control via ROS/inflammation reduction	Accelerated diabetic wound repair, angiogenesis, collagen deposition, re-epithelialization	[266]

### 4.6. Advanced Hydrogels

Hybrid hydrogels combining natural biopolymers with other natural or synthetic polymers have emerged as an effective strategy to overcome the limitations of single-component hydrogels. Combinations such as chitosan–alginate [320], chitosan–hyaluronic acid [321], gelatin–alginate [322], collagen–hyaluronic acid [274], cellulose–alginate [323], and silk fibroin–gelatin [324] integrate the complementary properties of each constituent, resulting in improved mechanical strength, structural stability, swelling behavior, injectability, and controlled biodegradation. The incorporation of synthetic polymers such as PVA [325], PEG [326], or Gelatin Methacryloyl (GelMA) hydrogels [327] further enhances printability, mechanical durability, and sustained therapeutic release while maintaining the biocompatibility of natural polymers. Hybrid hydrogels also provide multifunctional properties, including enhanced antimicrobial activity, antioxidant capacity, hemostasis, angiogenesis, and extracellular matrix remodeling, making them suitable for chronic, diabetic, infected, and highly exuding wounds [328]. Consequently, rational polymer selection and hybridization have become key design strategies for developing next-generation hydrogel wound dressings with improved therapeutic efficacy and clinical translation.

#### Intelligent Hydrogels for Next-Generation Wound Dressings

Recent advances in hydrogel technology have led to the development of self-healing, injectable, and conductive hydrogels, which address several limitations of conventional wound dressings. Stimuli-responsive hydrogels undergo physicochemical changes in response to endogenous or exogenous stimuli such as pH, temperature, glucose concentration, reactive oxygen species (ROS), enzymes, light, magnetic fields, or electric fields [329]. These systems enable on-demand release of antimicrobial agents, growth factors, anti-inflammatory drugs, or nucleic acids while simultaneously responding to pathological changes associated with infection, inflammation, or diabetic wounds. Such smart regulation minimizes systemic toxicity, prolongs therapeutic efficacy, and improves treatment. Self-healing hydrogels, formed through dynamic covalent or non-covalent interactions, can autonomously repair structural damage, thereby maintaining dressing integrity and extending functional lifespan under mechanical stress [330,331]. Their ability to restore integrity enhances durability, maintains a moist wound environment, and preserves therapeutic performance, making them particularly attractive for wounds located over highly mobile joints or irregular anatomical sites. Injectable hydrogels undergo in situ gelation after administration, enabling minimally invasive treatment, enable complete filling of irregular, deep, or tunneling wound cavities. Their excellent conformability, combined with sustained release of therapeutic agents and extracellular matrix-mimicking properties, supports cell infiltration, angiogenesis, and tissue regeneration while reducing patient discomfort during application [332]. Conductive hydrogels, fabricated by incorporating conductive polymers, carbon-based nanomaterials, or metallic nanostructures to recreate the endogenous bioelectrical environment involved in tissue repair [333]. By facilitating electrical signal transmission, these hydrogels promote fibroblast proliferation, keratinocyte migration, collagen deposition, angiogenesis, and nerve regeneration, making them particularly promising for chronic wounds, diabetic ulcers, and skin regeneration.

Emerging interdisciplinary technologies are accelerating the development of next-generation wound dressings. Three-dimensional bioprinting [334] enables patient-specific hydrogel fabrication, nanotechnology improves targeted and controlled therapeutic delivery, and AI-integrated wearable biosensors facilitate real-time wound monitoring and personalized treatment [335]. In addition, carrier-free supramolecular hydrogels [336], self-assembling systems, gene delivery platforms, and cell membrane- or exosome-functionalized hydrogels offer new opportunities for precision regenerative therapy [337]. Future research should focus on AI-assisted, patient-specific multifunctional hydrogels capable of real-time sensing, on-demand therapeutic delivery, and enhanced tissue regeneration, paving the way for personalized wound care.

## 5. Biopolymer Selection for Hydrogel Design

The clinical performance of hydrogel wound dressings is governed not only by their formulation but also by the intrinsic structural characteristics of the constituent biopolymers. The molecular architecture, functional groups, charge distribution, and degradation behavior of each polymer determine its swelling capacity, mechanical stability, bioactivity, and interaction with the wound microenvironment, making material selection a critical aspect of hydrogel design [5,16].

The selection of biopolymer hydrogels should be guided by the wound microenvironment, including the degree of inflammation, exudate volume, infection status, oxidative stress, and regenerative requirements (Table 13). Cellulose, chitosan, alginate, and hyaluronic acid possess distinct physicochemical and biological properties that make them suitable for different wound types. Chitosan is particularly advantageous for infected and chronic wounds because of its intrinsic antimicrobial, hemostatic, mucoadhesive, and immunomodulatory properties. However, its relatively poor solubility at physiological pH, variable deacetylation, and limited mechanical strength often necessitate chemical modification or blending with complementary polymers [23]. HA-based hydrogels are particularly suitable for diabetic chronic wounds, burn wounds, acute wounds, and other regenerative wound applications, owing to their ability to promote cell migration, angiogenesis, ECM remodeling, and re-epithelialization. Nevertheless, its rapid enzymatic degradation and weak mechanical properties generally require crosslinking to enhance stability in wound applications [244]. Similarly, alginate contains guluronic and mannuronic acid residues that readily form ionically crosslinked hydrogels with divalent cations, providing excellent exudate absorption and making alginate-based dressings particularly suitable for heavily exudative and bleeding wounds. For instance, calcium alginate can facilitate hemostasis through calcium-ion exchange and forms a moist gel upon contact with wound exudate. However, alginate may require reinforcement or combination with other polymers to improve mechanical strength, cellular adhesion, and performance in dry or minimally exudative wounds. In addition, their relatively limited cell-adhesive properties often require incorporation of bioactive molecules or combination with other natural polymers to enhance tissue regeneration [338]. The abundant hydroxyl groups in cellulose promote hydrogel formation and high water retention, enabling effective moisture regulation and exudate management while maintaining an optimal wound-healing environment. Owing to their excellent mechanical stability, transparency, and biocompatibility, cellulose-based hydrogels are well suited for acute wounds, burns, and moderately exudative wounds. In particular, bacterial cellulose exhibits high tensile strength, high purity, and excellent oxygen permeability, making it especially suitable for burns and chronic wounds requiring prolonged moisture retention. However, native cellulose is biologically inert and lacks intrinsic antimicrobial, antioxidant, or hemostatic activity. Consequently, cellulose-based hydrogels are frequently functionalized with bioactive agents, nanoparticles, antimicrobial compounds, or blended with other natural polymers to impart multifunctional therapeutic properties and accelerate tissue regeneration [28,312]. Despite their superior biological activity, their relatively rapid degradation, lower mechanical stability, and potential batch-to-batch variability frequently necessitate additional crosslinking or composite formulations [25,339,340,341]. These differences demonstrate that no single biopolymer fulfills all the requirements of an ideal wound dressing. Instead, rational hydrogel design increasingly relies on combining complementary polymers or developing multifunctional composite hydrogels to balance mechanical integrity, moisture retention, exudate management, antimicrobial activity, biodegradability, and biological functionality [24]. Consequently, polymer selection should be guided by wound-specific clinical requirements. Hydrogels with high fluid absorption capacity are preferable for heavily exuding chronic wounds, whereas highly bioactive materials that promote cell migration and angiogenesis are better suited for granulating or minimally exuding wounds. This design-oriented approach provides a stronger foundation for translating laboratory-developed hydrogels into clinically effective wound care products [6].

Recent advances in hydrogel-based wound dressings have expanded beyond conventional drugs and growth factors to include next-generation bioactive therapeutics. EVs such as exosomes have emerged as promising acellular therapeutics capable of modulating inflammation, promoting angiogenesis, and stimulating tissue regeneration through the delivery of proteins, lipids, and regulatory RNAs [342]. Similarly, nucleic acid therapeutics, including siRNA and mRNA, enable localized regulation of gene expression to suppress inflammation or enhance regenerative signaling, while clustered regularly interspaced short palindromic repeats/CRISPR-associated proteins (CRISPR/Cas)-based systems offer opportunities for precise gene editing to correct impaired healing pathways [343,344]. In addition, antimicrobial peptides have attracted considerable interest because of their broad-spectrum antimicrobial activity, low propensity for resistance development, and ability to promote tissue repair [345]. Hydrogel-mediated localized delivery of these bioactive agents provides sustained release, protects them from degradation, and minimizes systemic toxicity, representing a promising strategy for the treatment of chronic and infected wounds.

## 6. Patents

The patents included in this review were identified through systematic searches of major patent databases, including WIPO PATENTSCOPE, the United States Patent and Trademark Office (USPTO), Google Patents, and the European Patent Office (Espacenet). The literature search covered patents published up to May 2026. Search terms included combinations of “wound healing”, “hydrogel”, “biopolymer hydrogel”, “cellulose hydrogel”, “carboxymethyl cellulose”, “hydroxyethyl cellulose”, “hydroxypropyl methylcellulose”, “chitosan hydrogel”, “alginate hydrogel”, “hyaluronic acid hydrogel”, “wound dressing”, “tissue regeneration”, and “skin regeneration”. Patents were included if they described biopolymer-based hydrogel technologies intended for wound healing or wound dressing applications and reported novel compositions, fabrication methods, therapeutic strategies, or multifunctional wound care systems. Duplicate patent family members and patents unrelated to wound healing applications were excluded to ensure the relevance and uniqueness of the selected patents.

Recent patent trends highlight the growing interest in natural biopolymer-based hydrogels for wound healing applications, particularly those derived from chitosan, alginate, gelatin, HA, BC, cellulose derivatives, and natural gums. Patented formulations such as chitosan–acidic polysaccharide hydrogels (Patent No. 101897990), GelMa–alginate hydrogels for diabetic wound healing (Patent No. 201911046377), and crosslinked chitosan–hyaluronic acid hydrogel dressings (Patent Nos. WO/2022/132136 and 20210085823) demonstrate enhanced wound closure, antimicrobial activity, and improved tissue regeneration. Similarly, BC–alginate hydrogel biocomposites containing sodium fusidate (Patent No. 0002814059) and cellulose derivative-based hydrogels with high water absorption and elasticity (Patent No. 0002743941) have shown promising regenerative and wound dressing properties. Advanced multifunctional systems, including natural gum-based nanocomposite hydrogels incorporating silver nanoparticles (Patent No. 201931008616) and 3D-printed multi-biopolymer hydrogel constructs composed of alginate, gelatin, GelMa, cellulose, and chitosan (Patent Nos. WO/2022/197701 and 20240165296), further highlight the increasing focus on bioactive and mechanically stable wound dressings. In addition, biodegradable adhesive hydrogels (Patent No. 202331011069), HA-based hydrogels (Patent No. WO/2025/145252), and biodegradable drug-delivery hydrogels for wound and burn treatment (Patent No. WO/2023/129070) reflect the growing emphasis on multifunctional, smart, and clinically translatable hydrogel systems for advanced wound management. Recent patents on biopolymer-based hydrogels for wound healing applications are presented in Table 14. Critical analysis of recent patent activity indicates a clear shift from conventional moisture-retaining hydrogels toward multifunctional platforms integrating antimicrobial, antioxidant, adhesive, injectable, stimuli-responsive, and 3D-bioprinted technologies. While patents based on established biopolymers such as chitosan, alginate, hyaluronic acid, cellulose, and gelatin continue to dominate, the intellectual property landscape surrounding these materials has become increasingly saturated, prompting greater emphasis on novel crosslinking strategies, hybrid biopolymer composites, bioactive molecule incorporation, and advanced manufacturing approaches to establish patentability. This trend reflects the growing demand for clinically differentiated products with improved therapeutic performance and commercial value. Future innovation is therefore expected to focus on multifunctional, personalized, and scalable hydrogel platforms capable of addressing unmet clinical needs while overcoming existing intellectual property constraints and regulatory challenges [16,24,346].

## 7. Physicochemical Characterization of Hydrogel Wound Dressings

The therapeutic performance and clinical applicability of hydrogel wound dressings are governed not only by their biological activity but also by their physicochemical characteristics. Parameters including rheological behavior, crosslink density, swelling behavior, gel fraction, degradation kinetics, adhesive strength, injectability, fatigue resistance, sterilization stability, and long-term structural integrity determine the mechanical stability, drug release profile, degradation behavior, and interactions of hydrogels with the wound microenvironment (Table 15). Comprehensive characterization of these properties is therefore essential for optimizing hydrogel performance and facilitating successful clinical translation.

## 8. Clinical Translation and Current Wound Care Practice

### 8.1. Current Clinical Progress

Biopolymer-based hydrogels are well aligned with the fundamental principles of modern wound care by providing a moist wound environment, maintaining optimal moisture balance, absorbing excess wound exudate, and facilitating autolytic debridement while minimizing tissue trauma during dressing changes [16,18]. Their highly hydrated three-dimensional network promotes oxygen and nutrient diffusion, supports cell migration and proliferation, and mimics the extracellular matrix, thereby accelerating granulation tissue formation and re-epithelialization. Furthermore, incorporation of antimicrobial agents, metal nanoparticles, bioactive natural compounds, antimicrobial peptides, or growth factors enables effective infection control while reducing excessive inflammation and oxidative stress, which are major contributors to delayed wound healing [347,348]. Owing to their tunable swelling behavior and biocompatibility, biopolymer-based hydrogels are particularly suitable for the management of moderately to heavily exuding wounds, burns, pressure ulcers, venous leg ulcers, and diabetic foot ulcers, where maintaining an optimal wound microenvironment is essential for successful healing. Consequently, these hydrogels have emerged as versatile wound care platforms that integrate moisture management, infection control, exudate handling, and tissue regeneration within a single dressing system.

Current clinical trials highlight the growing translational potential of natural biopolymer-based hydrogels for wound healing applications, particularly those incorporating chitosan, alginate, collagen, and cellulose derivatives. Several interventional and phase II studies have evaluated the efficacy, safety, tolerability, and regenerative potential of these hydrogel systems in chronic wounds, diabetic foot ulcers, burns, and traumatic injuries. Chitosan-based formulations have shown promising outcomes in promoting wound healing, scar reduction, and patient recovery in chronic wounds and pediatric burn management (NCT04178525; NCT06987981). Alginate-containing hydrogels and CMC-based dressings have been investigated for diabetic foot ulcers and chronic traumatic wounds, demonstrating improved wound closure and moisture management (NCT03700580; NCT06978569). Collagen-based advanced skin substitutes combined with negative pressure wound therapy (NPWT) have also exhibited enhanced healing of full-thickness wounds (NCT06873867). Additionally, hydrogel dressings have been clinically assessed for cooling efficacy, cosmetic outcomes, local tolerability, and accelerated re-epithelialization (NCT06309446). These ongoing and completed clinical trials demonstrate the increasing clinical relevance of natural biopolymer-based hydrogels as advanced wound care systems with significant potential for chronic wound management and tissue regeneration. The clinical trial status of biopolymer-based hydrogels for wound healing applications is presented in Table 16. Although current clinical studies demonstrate encouraging improvements in wound healing, pain reduction, and patient tolerability, the overall clinical evidence remains limited by relatively small sample sizes, heterogeneous wound types, variable hydrogel formulations, and short follow-up periods [16]. Moreover, differences in treatment protocols and clinical endpoints make direct comparisons among studies challenging [349]. While most hydrogel dressings exhibit excellent biocompatibility and favorable safety profiles, long-term safety and effectiveness data, particularly for multifunctional hydrogels containing nanoparticles or growth factors, remain limited [346]. Therefore, adequately powered multicenter randomized controlled trials, standardized outcome measures, scalable Good Manufacturing Practice (GMP)-compliant manufacturing, and harmonized regulatory pathways are essential to support the successful clinical translation of advanced biopolymer-based hydrogel dressings [16,346].Despite extensive preclinical progress, only a limited number of hydrogel dressings have reached routine clinical use. Commercially available products, including IntraSite Gel, Purilon Gel, Nu-Gel, Aquaform, AmeriGel, Woulgan Gel, SANTYL, Solosite Gel, DuoDERM Hydroactive Gel, and Regranex, are primarily indicated for moist wound healing, autolytic debridement, and management of chronic wounds [16,349]. Clinical development is increasingly focused on advanced hydrogel systems incorporating mesenchymal stem cells, recombinant growth factors (e.g., erythropoietin, rhEGF, IGF-1), antimicrobial nanoparticles (silver, ZnO), anti-inflammatory agents (curcumin, sodium pentaborate, glycyrrhizic acid), and temperature-responsive or multifunctional biomaterials. These candidates are currently being evaluated from Phase I to Phase IV clinical trials, with several studies demonstrating encouraging safety and efficacy outcomes, particularly for diabetic foot ulcers and chronic wounds [346]. Nevertheless, despite encouraging clinical outcomes, most multifunctional hydrogel systems remain in the early stages of clinical development, highlighting the need to overcome several scientific, manufacturing, and regulatory challenges before widespread clinical adoption [24].

### 8.2. Barriers to Clinical Translation

Despite encouraging preclinical results, the clinical translation of advanced biopolymer hydrogels remains limited by scientific, manufacturing, and regulatory challenges. Discrepancies between in vitro and in vivo efficacy, the complexity of the wound microenvironment, limited high-quality clinical evidence, small patient cohorts, and non-standardized evaluation criteria hinder clinical validation. Furthermore, large-scale commercialization is challenged by batch-to-batch variability, quality control, sterilization, scalability, storage stability, and the high production costs of multifunctional hydrogels incorporating bioactive agents, cells, or nanomaterials.

Successful clinical translation of hydrogel wound dressings requires compliance with stringent regulatory and safety requirements in addition to demonstrating therapeutic efficacy. In the United States (US), hydrogel wound dressings are generally regulated by the U.S. Food and Drug Administration (FDA) as medical devices, with classification determined by the intended use, risk profile, and mechanism of action [350]. Regulatory approval also represents a major barrier to commercialization. In the United States, wound dressings are regulated by the US FDA as medical devices and are classified into Class I, II, or III according to risk, with most hydrogel dressings requiring 510(k) clearance or, for novel products, Premarket Approval (PMA). Multifunctional hydrogels incorporating drugs, biologics, antimicrobial agents, or living cells may be regulated as combination products, requiring additional evidence of safety, efficacy, manufacturing quality, and regulatory compliance. In contrast, under the European Union Medical Device Regulation (EU MDR 2017/745), devices are classified as Class I, IIa, IIb, or III based on intended use and patient risk, with greater emphasis on clinical evidence, post-market surveillance, risk management, and long-term safety evaluation [351]. Consequently, successful clinical translation requires not only optimization of hydrogel design and therapeutic performance but also standardized preclinical evaluation, robust multicenter clinical studies, scalable manufacturing processes, and early integration of regulatory requirements during product development. Many hydrogel-based wound dressings are classified as substance-based medical devices (SBMDs), for which regulatory qualification depends primarily on the primary mode of action (MoA). Under MDR Annex VIII, Rule 21 applies to SBMDs, whereas Rule 14 governs devices incorporating medicinal substances with an ancillary action. Distinguishing SBMDs from medicinal products remains a significant regulatory challenge, particularly for multifunctional hydrogels containing antimicrobial agents or bioactive substances, leading to classification inconsistencies across regulatory authorities [351]. Regardless of the regulatory pathway, biocompatibility evaluation should comply with the international organization for standardization (ISO) 10993 series, including assessment of cytotoxicity (ISO 10993-5), sensitization and irritation (ISO 10993-10), systemic toxicity, and, where appropriate, hemocompatibility and implantation (ISO 10993-1) [352]. Furthermore, compliance with Good Manufacturing Practice (GMP), sterilization validation, shelf-life stability, and post-market surveillance requirements is essential to ensure product quality, safety, and successful commercialization. As advanced multifunctional hydrogel systems continue to evolve, harmonized regulatory guidance and evidence-based classification frameworks will be essential to facilitate innovation while ensuring patient safety and regulatory consistency [351]. Key challenges limiting their widespread clinical adoption include inadequate mechanical durability, limited long-term safety data, batch-to-batch variability of natural polymers, high manufacturing costs, scalability, and complex regulatory requirements. Sterilization methods must be carefully optimized, as they may alter polymer structure, crosslinking density, swelling behavior, and mechanical properties [353]. For instance, gamma irradiation provides effective terminal sterilization but may induce polymer chain scission or additional crosslinking, altering mechanical properties and swelling behavior [354]. Ethylene oxide is suitable for heat-sensitive hydrogels but requires complete removal of residual gas, whereas steam sterilization is restricted to thermally stable formulations because it may promote polymer degradation [355,356]. Sterile filtration is applicable only to hydrogel precursor solutions before gelation. Therefore, sterilization methods should be selected according to polymer composition and crosslinking chemistry to maintain hydrogel performance while ensuring product safety and regulatory compliance. Moreover, robust quality control systems incorporating physicochemical characterization, sterility assurance, biocompatibility testing, stability evaluation, and GMP compliant production are essential to ensure product quality and reproducibility. Therefore, future efforts should focus on standardized manufacturing, robust long-term clinical evaluation, and well-defined regulatory pathways to accelerate commercialization of next-generation biopolymer-based hydrogel wound dressings [349].Future clinical translation of biopolymer-based hydrogels will depend on overcoming scientific, manufacturing, regulatory, and economic barriers through standardized fabrication methods, scalable GMP-compliant production, harmonized regulatory frameworks, and adequately powered multicenter clinical trials. Addressing these challenges will facilitate the commercialization and routine clinical adoption of next-generation multifunctional hydrogel wound dressings, ultimately bridging the gap between promising laboratory research and clinical practice.

## 9. Future Perspectives and Challenges

The next generation of biopolymer-based hydrogels is expected to evolve from passive wound dressings into intelligent therapeutic platforms capable of simultaneously sensing, monitoring, and responding to dynamic wound microenvironments.

Hydrogel-forming microneedles enable minimally invasive and localized delivery of therapeutic agents, including antibiotics, growth factors, nucleic acids, and stem cell-derived exosomes, thereby enhancing drug bioavailability while minimizing systemic adverse effects [357]. Smart and stimuli-responsive hydrogels or wearable wound sensors that respond to physiological triggers such as pH, temperature, glucose concentration, ROS, enzymes, electrical fields, and bacterial metabolites are attracting considerable attention because they enable on-demand drug release while minimizing systemic toxicity and improving therapeutic efficacy [18,358,359,360]. Such adaptive systems are particularly promising for chronic wounds, including diabetic foot ulcers, where continuous modulation of inflammation, oxidative stress, and infection is essential. Beyond these intelligent hydrogel platforms, several emerging interdisciplinary technologies are reshaping next-generation wound care. In parallel, artificial intelligence (AI)-enabled wound tracking utilizes computer vision and machine learning algorithms to objectively assess wound size, tissue characteristics, and healing progression, supporting clinical decision-making and remote patient monitoring [361]. Recent advances in three-dimensional (3D) bioprinting have expanded the applications of cellulose-, chitosan-, alginate-, and HA-based bioinks for fabricating patient-specific wound dressings and skin substitutes [362,363]. These bioinks provide excellent biocompatibility, tunable rheological properties, high water content, and ECM-mimicking environments that support cell viability, proliferation, and tissue regeneration. The integration of 3D and 4D bioprinting technologies is expected to facilitate the fabrication of patient-specific hydrogel dressings with precise architectures, controlled therapeutic delivery, and dynamic responsiveness to environmental stimuli, thereby improving tissue regeneration and personalized wound management [334,364]. Nanotechnology facilitates targeted delivery of drugs, plant bioactives, nucleic acids, exosomes, and antimicrobial agents while improving their stability and controlled release. Future hydrogel systems are also likely to become increasingly multifunctional through the incorporation of stem cell-derived EVs, antimicrobial peptides, growth factors, gene therapeutics, and nanomaterials, enabling simultaneous antimicrobial, anti-inflammatory, angiogenic, immunomodulatory, and regenerative functions [365]. Another promising direction is the development of wearable hydrogel-based biosensing systems capable of continuously monitoring wound biomarkers, including pH, temperature, glucose, oxygen, and bacterial infection, while enabling real-time therapeutic intervention and remote patient monitoring [366,367]. Another emerging strategy is the development of carrier-free hydrogels, formed by the self-assembly of therapeutic molecules without conventional carriers [51,368]. For instance, the natural ellagitannin β-D-penta-O-galloyl glucose (β-D-PGG) spontaneously self-assembles into nanofiber hydrogels through hydrogen bonding and π–π stacking [336]. The resulting hydrogel exhibits antibacterial and anti-inflammatory activities by promoting M2 macrophage polarization, making it a promising candidate for wound healing. These hydrogels provide high drug loading, sustained release, structural support, and therapeutic activity with improved biocompatibility and biodegradability. Despite their promise for next-generation wound dressings, challenges in large-scale production, reproducibility, and regulatory approval remain.

Although biopolymer hydrogels have demonstrated excellent biocompatibility and regenerative potential, their clinical translation requires comprehensive safety evaluation. Important considerations include cytocompatibility, immunogenicity, biodegradation behavior, sterilization methods, residual crosslinking agents, batch-to-batch variability, and long-term biosafety. Many natural biopolymers exhibit relatively poor mechanical strength, rapid degradation, and variable physicochemical properties, which may compromise their long-term performance in highly exudative or load-bearing wounds [16,94]. The incorporation of bioactive molecules, nanoparticles, or synthetic polymers may further alter toxicity profiles and should therefore be evaluated through standardized in vitro, in vivo, and clinical safety studies. In addition, batch-to-batch variability arising from natural sources, cytocompatibility, potential immunogenicity associated with impurities, limited long-term biosafety data, and difficulties in sterilization, storage, and large-scale manufacturing remain significant barriers to commercialization. These challenges may be addressed through hybrid hydrogel design combining natural and synthetic polymers, advanced crosslinking strategies, incorporation of reinforcing nanomaterials, standardized extraction and manufacturing protocols, and implementation of quality-by-design (QbD) principles to improve reproducibility and product consistency [16,359,369]. However, the inclusion of nanoparticles and therapeutic molecules can also lead to toxicity concerns and regulatory complexities. Moreover, most studies remain limited to in vitro and preclinical investigations, while well-designed multicenter clinical trials are still insufficient to fully establish long-term efficacy and safety. Future efforts should focus on optimizing hydrogel formulations, improving manufacturing scalability and reproducibility, establishing standardized evaluation protocols, and conducting robust long-term preclinical and clinical studies to facilitate regulatory approval, commercialization, and widespread clinical adoption of next-generation biopolymer-based hydrogel wound dressings.

## 10. Conclusions

Biopolymer-based hydrogels serve as a versatile and effective category of wound dressing material for the treatment of acute and chronic wounds. Natural biopolymers including cellulose and its derivatives, chitosan, alginate, and HA offer good biocompatibility, biodegradability, moisture retention, and bioactivity to facilitate various phases of wound healing. Recent advancements in smart hydrogels, nanocomposite systems, injectable formulations, and bioactive-loaded hydrogel platforms have further enhanced antimicrobial activity, hemostatic performance, tissue regeneration, angiogenesis, and controlled therapeutic delivery [14,16]. In addition, growing patent activity and ongoing clinical trials highlight the increasing translational and commercial potential of these systems for advanced wound management. Overall, the continuous evolution of multifunctional hydrogel technologies, combined with emerging fabrication strategies such as 3D bioprinting and stimuli-responsive design, is expected to revolutionize wound care and skin tissue engineering. However, overcoming current limitations related to mechanical strength, biosafety, scalability, regulatory approval, and long-term clinical validation remains essential for successful clinical implementation. With continued interdisciplinary research and clinical development, biopolymer-based hydrogels are anticipated to play a major role in next-generation personalized wound healing therapies.

## Figures and Tables

**Table 1:** Comparison of representative review articles on biopolymer hydrogels for wound healing published during the last five years.

Biopolymers Covered	Main Focus	Key Limitations	Unique Contribution of the Review	Reference
Cellulose derivatives	Cellulose derivative-based wound dressings, including properties, wound healing mechanisms, formulation strategies, and biomedical applications.	Limited to cellulose derivatives; limited discussion of other biopolymers, comparative analysis, clinical translation, and emerging technologies.	Comprehensive evaluation of cellulose derivative dressings, highlighting the relationship between polymer structure, functional properties, and wound-healing performance.	[20]
Chitosan	Chitosan-based hydrogels for wound healing, including properties, fabrication, bioactivity, and therapeutic applications.	Emphasizing chitosan; limited discussion of other biopolymers, clinical translation, safety, and emerging technologies.	Comprehensive overview of chitosan hydrogel design, functional modification, and wound-healing mechanisms.	[25]
Hyaluronic acid	Role of hyaluronic acid in skin aging and wound healing, including biological functions, molecular mechanisms, and therapeutic applications.	Centered on hyaluronic acid; limited coverage of other biopolymers, advanced hydrogel design, and clinical translation.	Extensive discussion of hyaluronic acid biology, emphasizing its molecular weight-dependent functions in skin regeneration, inflammation, ECM remodeling, and wound repair.	[26]
Sodium alginate	Sodium alginate-based biodegradable dressings for skin wound healing, including properties, fabrication, and therapeutic performance.	Focused only on alginate; limited coverage of other biopolymers, advanced hydrogel technologies, and clinical translation.	Systematic evaluation of alginate-based dressings, highlighting their biodegradability, bioactivity, and effectiveness in skin wound healing.	[27]
Chitosan and cellulose	Chitosan- and cellulose-based hydrogels for wound management, including preparation, properties, drug delivery, and therapeutic applications.	Restricted to two biopolymers; limited discussion of molecular mechanisms, clinical translation, and emerging technologies.	Comparative overview of chitosan- and cellulose-based hydrogel systems and their potential as wound dressings and therapeutic carriers.	[28]
Collagen, chitosan, alginate, and other hydrogels	Recent advances in hydrogel-based wound dressings for skin repair, including material properties, fabrication, and therapeutic applications.	Broad overview with limited mechanistic analysis, clinical translation, safety, and emerging technologies.	Extensive summary of natural hydrogel materials and recent advances in their application for skin repair and wound dressing.	[29]

**Table 2:** Comparative overview of various types of cellulose used in wound healing applications.

Characteristics	Bacterial Cellulose	Nanocellulose	Oxidized Cellulose	Cellulose Nanocrystals
Source	Produced by bacteria such as *Komagataeibacter xylinus*	Derived from plant cellulose or bacterial cellulose	Chemically modified cellulose through oxidation	Obtained by acid hydrolysis of cellulose fibers
Structure	Highly pure three-dimensional nanofibrous network	Nanoscale fibrillar cellulose structure	Cellulose containing carboxyl or aldehyde functional groups	Highly crystalline rod-like nanoparticles
Purity	Very high purity, free from lignin and hemicellulose	Depends on source and purification process	Depends on degree of oxidation	High crystallinity and purity
Biocompatibility	Excellent	Excellent	Excellent	Excellent
Biodegradability	Biodegradable	Biodegradable	Biodegradable	Biodegradable
Water-holding capacity	Very high	High	Moderate to high	Moderate
Mechanical strength	Excellent tensile strength and flexibility	High mechanical strength	Moderate mechanical strength	Very high rigidity and strength
Hemostatic property	Moderate	Limited	Excellent hemostatic activity	Limited
Antimicrobial property	Can be enhanced by loading bioactives	Can be functionalized with antimicrobial agents	Mild intrinsic antimicrobial effect due to acidic nature	Usually requires functionalization
Moisture retention	Excellent	Excellent	Good	Moderate
Transparency	High transparency in hydrogel/film form	Variable	Moderate	Good
Drug loading ability	Excellent	Excellent	Good	High surface area favors drug adsorption
Major advantages in wound healing	Maintains moist environment, promotes tissue regeneration	Enhances mechanical stability and scaffold performance	Promotes hemostasis and bioresorption	Reinforces wound dressings and improves mechanical properties
Limitations	High production cost	Aggregation tendency	Lower mechanical flexibility	Brittleness at high concentration
Common wound dressing forms	Hydrogels, films, membranes, scaffolds	Hydrogels, nanofibers, films, composites	Absorbable hemostatic dressings, gauze	Composite films, hydrogels, nanocomposites

**Table 3:** Carboxymethyl cellulose (CMC)-based wound healing formulations and their composition, functions, mechanistic outcomes, design strategy, and translational stage.

Composition	Wound Healing Functions	Mechanistic/Functional Outcomes	Wound Type	Hydrogel Design Strategy	Translation Stage	References
Superoxide dismutase, recombinant human epidermal growth factor	Antioxidant activity, angiogenesis promotion, tissue regeneration	Enhanced cell migration and proliferation, reduced DNA damage, shortened inflammatory phase, increased collagen deposition and angiogenesis.	Diabetic wound	Growth factor-loaded antioxidant hydrogel	In vitro + In vivo	[100]
Nitric oxide	Antimicrobial activity, angiogenesis enhancement, vascular regeneration	Sustained nitric oxide release, promoted angiogenesis/vascular reconstruction, and antibacterial activity against *Staphylococcus aureus* (*S. aureus).*	Infected wound	Gas-releasing hydrogel	In vitro + In vivo	[101]
Berberine	Anti-inflammatory, antioxidant, anti-hyperproliferative	Reduced oxidative stress and inflammation, decreased keratinocyte hyperproliferation (~25%).	Psoriatic/chronic wound	Bioactive phytochemical-loaded hydrogel	In vitro + In vivo	[102]
Tannic acid	Antioxidant, antibacterial, hemostatic, tissue regeneration	Strong adhesion, self-healing, and injectability, reduced inflammation and oxidative stress, promoted neovascularization.	Full-thickness wound	Bioadhesive self-healing hydrogel	In vitro + In vivo	[103]
None (intrinsic polymer activity)	Moisture retention, antibacterial, antioxidant, promotes regeneration	Rapid in-situ gelation (~30 s), high swelling capacity (~10×), biocompatible, accelerates tissue regeneration.	Full-thickness diabetic wound	Intrinsically bioactive in hydrogel	In vitro + In vivo	[104]
Fucoidan (*Ecklonia cava*)	Antioxidant, anti-inflammatory, promotes collagen synthesis and cell migration	Sustained fucoidan release, ROS scavenging, reduced nitric oxide (NO), increased collagen production, enhanced re-epithelialization.	Full-thickness wound	Marine polysaccharide-based hydrogel	In vitro + In vivo	[105]
L-carnosine	Promotes wound healing, biocompatible, hemocompatible	Sustained carnosine release (>80% in 48 h), supports cell viability, accelerates wound closure and tissue regeneration.	Excisional wound (rat model)	Drug-loaded hydrogel	In vitro + In vivo	[106]
Curcumin	Antibacterial, anti-inflammatory, promotes wound healing, reduces scar formation	pH-responsive controlled curcumin release, high swelling (~1300%) to maintain a moist environment, good biocompatibility, accelerated wound closure, reduced inflammation and minimized scar formation in vivo.	Full-thickness wound	pH-responsive drug delivery hydrogel	In vitro + In vivo	[107]

**Table 4:** Representative hydroxyethyl cellulose (HEC)-based hydrogels for wound healing and their composition, functions, outcomes, design strategy, and translational stage.

Composition	Wound Healing Functions	Mechanistic/Functional Outcomes	Wound Type	Hydrogel Design Strategy	Translation Stage	References
Collagen plus naproxen	Structural support, exudate management, smart monitoring	Improved flexibility and peelability, enhanced mechanical strength, high swelling capacity, pH-responsive color change, controlled biphasic drug release (>75%/24 h).	Burn wound	pH-sensitive composite hydrogel film	In vitro	[139]
Silk nanofiber-Mg^2+^ complex, glycerol	Angiogenesis, tissue regeneration, anti-inflammatory	Skin-like elasticity, enhanced cell proliferation and migration, increased collagen deposition, reduced scarring, accelerated diabetic wound healing.	Diabetic wound	Natural polymer/ composite hydrogel	In vivo	[140]
Fibroblasts	Cellular regeneration, tissue repair, ECM deposition	Enhanced fibroblast activity, increased cell proliferation, improved granulation tissue formation, angiogenesis, re-epithelialization.	Full-thickness wound	Cell-laden hydrogel	In vitro + In vivo	[141]
Hyaluronic acid, gelatin, tetracalcium phosphate	Tissue regeneration, moisture retention, angiogenesis support	Rapid wound closure (up to 100%/24 h), enhanced cell migration, angiogenesis, antibacterial activity, high swelling capacity.	Full-thickness wound	Composite hydrogel scaffold	In vitro + In vivo	[142]
Zinc oxide	Antimicrobial, regenerative	Accelerated burn healing, ~24% higher healing rate, faster wound closure compared to controls.	Burn wound	Biopolymer-stabilized inorganic metal nanoparticle mixed hydrogel	In vivo	[143]
Zinc oxide nanoparticles, menthol (encapsulated in cationic β-cyclodextrin)	Antimicrobial, analgesic, controlled drug release, biocompatibility, moisture retention	Uniform nanoparticle dispersion enhanced antibacterial activity, while cyclodextrin-mediated menthol encapsulation enabled sustained release. The hydrogel exhibited excellent mechanical strength, swelling, water retention, oxygen permeability, and biocompatibility.	Infected wound	Nanoparticle-incorporated in composite hydrogel	In vitro	[144]
*Curcuma longa* extract	pH-responsive wound monitoring, controlled drug release, exudate management	Exhibited distinct color change (yellow at pH ≤ 7, dark red at pH > 7) enabling visual wound status monitoring, tunable porous structure (370–100 µm) and high swelling capacity (up to 1839%) supported exudate absorption, pH-dependent release with higher release at pH 7.4 and sustained release at alkaline pH (8 ± 0.2).	Chronic wound	Stimuli-responsive hydrogel	In vitro	[145]
Zinc acetate (Zn^2+^) in hydroxyethylcellulose/ polyvinyl alcohol cellulose film	Anti-inflammatory, anti-apoptotic, angiogenesis, tissue remodeling	Enhanced wound contraction and histological recovery, reduced pro-inflammatory cytokines (IL-1β, TNF-α), increased collagen I and Bcl-2 levels, early downregulation (day 8) followed by upregulation (day 16) of VEGF, TGF-β, MMP2, and TIMP2, indicating controlled inflammation and improved tissue remodeling.	Burn wound	Metal ion-based composite hydrogel film	In vivo	[146]

**Table 5:** Selected hydroxypropyl methylcellulose (HPMC)-based hydrogels for wound healing and their composition, functions, mechanistic outcomes, design strategy, and translational stage.

Composition	Wound Healing Functions	Mechanistic/Functional Outcomes	Wound Type	Hydrogel Design Strategy	Translational Stage	References
Ofloxacin, micronized biodegradable suture fibres (polyglycolic acid, Vicryl^®^, catgut)	Antibacterial, moisture retention, structural support, enhanced wound healing	Broad-spectrum antimicrobial action, controlled drug release, high swelling capacity (531.8–1700%), improved flexibility (70–120% elongation), ~95% wound closure in 14 days, increased collagen deposition and minimal scar formation, no drug-excipient interaction.	Full-thickness/infected wound	Drug-loaded composite hydrogel reinforced with biodegradable suture	In vitro + In vivo	[171]
Hyaluronic acid, glycerol, copper-iron layered double hydroxide (CuFe-LDH), hydroxyapatite, Carboxymethyl chitosan	Thermosensitive gel formation, antibacterial activity, biomineralization, hemostasis, tissue regeneration	Injectable thermosensitive scaffold with reduced gelation time, strong antibacterial activity against *S. aureus* and *E. coli*, enhanced biomineralization and osteoconductivity, rapid hemostasis, and improved wound healing and tissue repair.	Infected wound	Injectable multifunctional thermosensitive composite hydrogel	In vitro	[172]
Hypromellose succinate-crosslinked chitosan with 1-ethyl-3-(3-dimethylaminopropyl) carbodiimide/ N-hydroxysuccinimide crosslinking system, gentamycin sulfate	Wound dressing, antibacterial activity, tissue regeneration, sustained drug delivery	Chemically crosslinked hydrogel with enhanced mechanical strength, high swelling, optimal water vapor and oxygen permeability, good biocompatibility, and sustained gentamycin release for prolonged antibacterial activity.	Infected wound	Crosslinked antibacterial hydrogel	In vitro	[173]
Carboxymethyl starch, zinc oxide nanoparticles	Wound dressing, antibacterial activity, moisture retention, enhanced permeability, tissue healing support	Hydrogen bonding improved film integrity, while ZnO nanoparticles enhanced antibacterial and physicochemical performance.	Full-thickness wound	Nanoparticle-incorporated porous nanocomposite hydrogel films	In vitro	[174]
Carboxymethyl starch, succinic acid, gallic acid	Wound dressing, antibacterial activity, enhanced mechanical strength, moisture retention, tissue healing support	Hydrogen bonding and crosslinking improved structural integrity, mechanical strength, swelling, and moisture regulation, while gallic acid provided antibacterial activity, collectively promoting wound healing.	Full-thickness wound	Crosslinked porous composite bioactive hydrogel films	In vitro	[175]
Coumestrol complexed with hydroxypropyl-β-cyclodextrin	Wound healing, cell proliferation, tissue regeneration, enhanced drug solubility and delivery	Cyclodextrin improved coumestrol solubility and topical delivery, enhancing fibroblast activity, accelerating wound closure (~50%), and promoting tissue repair.	Excisional wound	Cyclodextrin-based drug delivery hydrogel	In vitro + In vivo	[176]
HPMC-capped copper nanoparticles	Antibacterial activity, wound dressing, infection control, tissue healing support	Nanocomposite HPMC hydrogel provided enhanced structural stability, antibacterial activity against *S. aureus* and *E. coli*, biocompatibility, and effective wound healing.	Infected wound	Nanoparticle-loaded nanocomposite hydrogel films	In vitro	[177]

**Table 6:** Chitosan-based formulations for wound healing, highlighting their composition, therapeutic functions, mechanistic outcomes, design strategy, and translational stage.

Composition	Wound Healing Functions	Mechanistic/Functional Outcomes	Wound Type	Hydrogel Design Strategy	Translational Stage	References
Stem cells from human exfoliated deciduous teeth, Chitosan/palmitoyl tetrapeptide-7 hydrogel	Multifunctional wound healing (antibacterial, anti-inflammatory, angiogenic, regenerative)	Promoted > 95% wound closure by enhancing angiogenesis, M2 macrophage polarization, collagen deposition, and anti-inflammatory cytokine modulation in diabetic wounds.	Diabetic wound	Stem cell-loaded injectable polypeptide hydrogel	In vitro + In vivo	[211]
Oxidized *Ganoderma lucidum* polysaccharide, CMCS hydrogel	Hemostatic, anti-inflammatory, antioxidant, angiogenic, and regenerative wound healing	Promoted hemostasis, M2 macrophage polarization, angiogenesis, collagen deposition, and accelerated wound healing.	Acute bleeding/ full-thickness wound	Polysaccharide-based bioactive hydrogel	In vitro + In vivo	[212]
Vancomycin, ciprofloxacin, amoxicillin, Chitosan-alginate polyelectrolyte complex hydrogel	Antibacterial, controlled drug delivery, infection management	Alginate/CaCl_2_ crosslinking enabled pH-responsive antibiotic release, enhancing antibacterial efficacy, structural stability, biocompatibility, and swelling.	Infected wound	Antibiotic-loaded polyelectrolyte complex hydrogel	In vitro	[213]
Amoxicillin-loaded copper oxide nanoparticle, CMCS/starch nanocomposite hydrogel	Antibacterial, controlled drug delivery, infection management	In situ CuO nanoparticles enhanced antibacterial activity, pH-responsive swelling, and sustained drug release, providing prolonged therapeutic efficacy.	Infected wound	Nanoparticle-loaded nanocomposite hydrogel	In vitro	[214]
Amikacin, Crosslinked chitosan-folic acid hydrogel film	Antibacterial, controlled drug release, wound protection	Sustained amikacin release with low burst effect, potent antibacterial activity, good cytocompatibility, and stable film properties, enabling prolonged infection control and wound healing.	Infected wound	Crosslinked drug-loaded hydrogel film	In vitro	[215]
Aloe vera and tetrasodium ethylenediamine-tetraacetic acid, β-glycerophosphate-crosslinked chitosan hydrogel	Antibacterial, anti-inflammatory, wound healing, biocompatible scaffold	Porous, highly swollen hydrogel maintained a moist wound environment, exhibited antibacterial activity against *S. aureus* and *P. aeruginosa*, and promoted tissue regeneration with excellent hemocompatibility and cytocompatibility.	Infected wound	Crosslinked thermosensitive bioactive hydrogel	In vitro + In vivo	[216]
Resveratrol micelles, chitosan hydrochloride, HEC, and β-glycerol phosphate	Antioxidant, antibacterial (*S. aureus*), anti-inflammatory, promotes angiogenesis, enhances collagen deposition, hemostatic	Sustained RES release reduces oxidative stress, inhibits drug-resistant bacteria, modulates inflammation, promotes neovascularization and ECM formation, enables rapid hemostasis, supports biocompatibility and tissue regeneration.	Chronic wound	Phytochemical coated polymer scaffold (mat)	In vitro + In vivo	[217]
Phellinus igniarius polysaccharides, L-arginine-conjugated chitosan	Antibacterial, antioxidant, anti-inflammatory, promotes angiogenesis, enhances collagen deposition, accelerates diabetic wound healing	Promoted antibacterial activity, epithelial regeneration, collagen deposition, angiogenesis, and oxidative stress reduction by modulating NF-κB and HIF-1α signaling, accelerating diabetic wound healing.	Diabetic wound	Bioactive polysaccharide-conjugated hydrogel	In vitro + In vivo	[218]
Doxycycline hyclate, Crosslinked CMCS and aldehyde hyaluronic acid	Broad-spectrum antibacterial, controlled drug delivery, reduces infection, improves local drug retention, minimizes systemic side effects	Controlled drug release via Fickian diffusion enhanced local drug retention and antibacterial efficacy, while optimized physicochemical properties supported sustained delivery and improved wound coverage.	Infected wound	Crosslinked drug delivery hydrogel	In vitro + Ex vivo	[219]

**Table 7:** Multifunctional hyaluronic acid-based hydrogels for wound healing: key components, functional properties, healing outcomes, design strategy, and translational stage.

Type of Multifunctional Hydrogel	Key Components/Modification	Functional Properties	Wound Healing Outcomes	Wound Type	Hydrogel Design Strategy	Translational Stage	References
Photo-responsive supramolecular HA hydrogel	Azobenzene and β-cyclodextrin host-guest interactions with epidermal growth factor (EGF)	Light-triggered dynamic crosslinking and controlled epidermal growth factor release	Enhanced granulation tissue formation, angiogenesis, and growth factor expression	Full-thickness wound	Photo-responsive hydrogel	In vitro + In vivo	[265]
Glucose-responsive HA hydrogel	HA methacrylate- phenylboronic acid-responsive hyaluronic acid derivative and catechin via borate ester bonding	Glucose-responsive drug release, antioxidant activity, ROS scavenging	Reduced inflammation, enhanced angiogenesis, accelerated diabetic wound healing	Diabetic wound	Glucose-responsive hydrogel	In vitro + In vivo	[266]
Electro-responsive HA click hydrogel	Conductive polymer poly(hydroxymethyl-3,4-ethylenedioxythiophene)	Increased porosity, mechanical strength, electrochemical activity	Enhanced epithelial cell migration and rapid wound closure	Full-thickness wound	Electro-responsive conductive hydrogel	In vitro	[263]
Multifunctional nanocomposite hydrogel	Modified HA, plant extract, and in situ-synthesized nanosilver	Self-healing, adhesive, antioxidant, pH/enzyme-responsive antibacterial activity	Reduced inflammation, enhanced cell proliferation, accelerated healing of resistant infections	Infected wound	Dual stimuli-responsive self-healing nanocomposite hydrogel	In vitro + In vivo	[267]
Sulfonium-crosslinked HA polymer	Sulfonium-crosslinked HA with antibiotic loading	Antimicrobial activity, hyaluronidase-responsive antibiotic release, biofilm eradication	Restored antibiotic efficacy against MRSA, VRE, and *P. aeruginosa* and improved wound healing	Infected wound	Enzyme-responsive self-healing antibacterial hydrogel	In vitro + In vivo	[264]

**Table 8:** Hyaluronic acid-based wound healing formulations and their composition, functions, mechanistic outcomes, design strategy, and translational stage.

Composition	Wound Healing Functions	Mechanistic/Functional Outcomes	Wound Type	Hydrogel Design Strategy	Translational Stage	References
Salecan, polyhexamethylene biguanide	Antimicrobial, self-healing, injectable, anti-inflammatory, hemostatic, promotes tissue regeneration	pH/hyaluronidase-responsive degradation, sustained drug release, inhibition of *S. aureus* and *E. coli*, reduced inflammation, enhanced collagen deposition, angiogenesis, and accelerated wound closure.	Infected wound	Stimuli-responsive injectable hydrogel	In vitro + In vivo	[278]
Tea polyphenol-stabilized silver nanoparticles	Antioxidant, anti-inflammatory, antibacterial, promotes tissue regenerative	Glucose-responsive nanoparticle release promoted ROS scavenging, antibacterial activity against *E. coli* and *S. aureus*, angiogenesis, collagen deposition, and accelerated wound healing.	Diabetic wound	Glucose-responsive nanocomposite hydrogel	In vitro + In vivo	[279]
Gallic acid	Biocompatibility enhancement, anti-inflammatory, tissue regenerative	Controlled drug release, improved mechanical stability, enhanced fibroblast proliferation and cell viability, optimal pore structure for cell growth, and promotion of skin tissue regeneration.	Full-thickness wound	Bioactive enriched hybrid scaffold hydrogel	In vitro	[280]
Resveratrol	Antioxidant, anti-aging, promotes collagen synthesis, enhances skin regeneration	CD44-targeted delivery enhanced skin penetration, drug deposition, collagen production, and cellular repair while reducing ROS.	Skin wound/Photoaged skin	CD44-targeted modified niosome-based hydrogel	In vitro	[281]
ε-polylysine	Antibacterial, biocompatible, exudate absorption, promotes tissue healing	Broad-spectrum antibacterial activity, enhanced mechanical strength and elasticity, accelerated degradation and drug release, high exudate absorption, improved moisture balance, and enhanced wound healing environment.	Exuding wound	Nanofiber mat-based absorbent antibacterial hydrogel	In vitro	[282]
Mupirocin	Antibacterial, antioxidant, hemostatic, promotes tissue regeneration	ROS scavenging, inhibition of *E. coli* and *S. aureus*, reduced inflammation (↓ TNF-α), enhanced angiogenesis (↑ CD31), improved cell migration, controlled drug release, and accelerated wound healing.	Infected wound	ROS-responsive composite antibacterial hydrogel	In vitro + In vivo	[63]
Zinc oxide	Antibacterial, anti-inflammatory, promotes tissue regeneration	Sustained ZnO release inhibited *E. coli* and *S. aureus*, reduced inflammation, and accelerated burn wound healing.	Burn wound	Nanoparticles loaded in electrospun nanofibrous dressings in composite hydrogels	In vitro + In vivo	[283]
Silver sulfadiazine	Antibacterial, anti-inflammatory, promotes tissue regeneration	Reduced bacterial load (including biofilms), suppressed inflammation, enhanced re-epithelialization, and accelerated wound closure.	Burn wound and other infected wound	Drug-loaded antibacterial hydrogels	In vitro + In vivo	[284]

**Table 13:** Comparative applicability of major biopolymer hydrogels for different wound types and microenvironment-guided material selection.

Biopolymer	Most Suitable Wound Types	Key Microenvironment Characteristics	Major Advantages	Main Limitations	Material Selection Principle
Cellulose	Acute wounds, burn wounds, moderately exudative wounds	Moderate inflammation, low-to-moderate exudate, minimal infection	Excellent moisture regulation, mechanical strength, transparency, oxygen permeability, biocompatibility, high water retention	Lacks intrinsic antimicrobial, antioxidant, and hemostatic activity	Preferred for structural support, moisture retention, and exudate management; functionalization is required for infected or chronic wounds.
Chitosan	Infected wounds, diabetic chronic wounds, bleeding wounds	High inflammation, microbial burden, moderate exudate, oxidative stress	Intrinsic antimicrobial, hemostatic, mucoadhesive, anti-inflammatory, immunomodulatory properties	Poor solubility at physiological pH, limited mechanical strength, variable degree of deacetylation	Preferred for infected and highly inflamed wounds requiring antimicrobial protection, hemostasis, and immune modulation.
Alginate	Highly exudative wounds, bleeding wounds, infected wounds with excessive exudate	Heavy exudate, bleeding, moderate infection	Excellent fluid absorption, rapid gel formation, hemostatic activity, moist wound environment	Poor cell adhesion, weak mechanical stability, less suitable for dry wounds	Preferred when rapid exudate absorption, gel formation, and bleeding control are the primary requirements.
Hyaluronic acid	Acute wounds, diabetic chronic wounds, burn wounds, regenerative wounds	Persistent inflammation, oxidative stress, impaired angiogenesis, ECM damage	Promotes cell migration, angiogenesis, ECM remodeling, re-epithelialization, excellent hydration and biocompatibility	Rapid enzymatic degradation, weak mechanical strength, lacks intrinsic antimicrobial activity	Preferred for wounds requiring tissue regeneration and ECM restoration; often combined with other biopolymers or bioactive agents to improve stability and antimicrobial performance.

**Table 14:** Recent patents on biopolymer-based hydrogels incorporating diverse active agents for wound healing, including publication number, date of publication, patent jurisdiction, title, summary of invention, and corresponding reference.

Publication Number	Date of Publication	Patent Jurisdiction	Title	Type of Biopolymer Used	Summary of Invention	Reference
101897990	6 July 2010	China	Dressing composition used for inhibiting scars and accelerating wound healing and application thereof	Chitosan and acidic polysaccharides	Natural polysaccharide-based hydrogel dressing containing chitosan, acidic polysaccharides, and Panax notoginsenosides with antibacterial and regenerative properties.	CN101897990A
20170224874	1 July 2015	United States of America	Hydrogels for treating and ameliorating wounds	Hydrogel polymer (unspecified source)	Hydrogel-based pharmaceutical composition containing skin grafts.	US20170224874A1
201931008616	5 March 2019	India	Natural gum-based nanocomposite hydrogel	Natural gum-based biopolymers	Nanocomposite hydrogel incorporating silver nanoparticles for wound healing.	IN201931008616A
201911046377	14 November 2019	India	Wound dressing hydrogel and process for preparation thereof	Gelatin methacrylamide (GelMa) and alginate	GelMa–alginate hydrogel containing TDFA for diabetic wound healing.	IN201911046377A
0002743941	1 March 2021	Russian Federation	Method for producing biopolymeric hydrogel	Cellulose derivatives	Crosslinked cellulose derivative-based hydrogel.	RU2743941C1
20210085823	25 March 2021	United States of America	Methods of making chitosan/hyaluronic acid hydrogel compositions	Chitosan and hyaluronic acid	Crosslinked chitosan–hyaluronic acid hydrogel wound dressing.	US20210085823A1
WO/2022/132136	23 June 2022	PCT (International)	Methods of making chitosan/hyaluronic acid hydrogel compositions	Chitosan and hyaluronic acid	Crosslinked chitosan–hyaluronic acid hydrogel wound dressing.	WO2022132136A1
WO/2022/197701	22 September 2022	PCT (International)	Dressings and methods for wound healing	Alginate, gelatin, GelMa, cellulose, and chitosan	3D-printed BBG-based hydrogel construct with multiple biopolymers.	WO2022197701A1
202331011069	17 February 2023	India	Formulation of adhesive and wound healing dual active hydrogel with molecules isolated from biological sources	Biodegradable polymer hydrogel (unspecified source)	Biodegradable adhesive hydrogel designed for wound healing, exhibiting biocompatibility, low toxicity, effective adhesion to wet tissues, and potential for localized drug delivery and enhanced tissue repair.	IN202331011069A
0002814059	3 May 2023	Russian Federation	Method of producing biocomposite materials with regenerative and antiseptic properties based on bacterial cellulose hydrogels	Bacterial cellulose and alginate	Bacterial cellulose–alginate hydrogel biocomposite containing sodium fusidate.	RU2814059C1
WO/2023/129070	6 July 2023	PCT (International)	Biocompatible and biodegradable hydrogels suitable for use in wound and burn treatment	Biodegradable biopolymer hydrogel (unspecified source)	Biocompatible biodegradable hydrogel with controlled drug delivery capability.	WO2023129070A1
20240165296	23 May 2024	United States of America	Dressings and methods for wound healing	Alginate, gelatin, GelMa, cellulose, and chitosan	3D-printed BBG-biopolymer hydrogel construct for wound healing.	US20240165296A1
WO/2024/136811	27 June 2024	PCT (International)	Wound healing gel formulation comprising *Melaleuca viridiflora* L. and *Boswellia carterii* L.	Hydrogel polymer (unspecified source)	Hydrogel formulation containing phytotherapeutic oils to enhance wound healing.	WO2024136811A1
WO/2025/145252	10 July 2025	PCT (International)	Biocompatible hyaluronic acid hydrogels	Hyaluronic acid	Urethane-crosslinked hydrogel based on hyaluronic acid.	WO2025145252A1

**Table 15:** Key physicochemical characterization parameters of hydrogel wound dressings and their influence on biological performance.

Parameter	Significance	Influence on Biological Performance	Representative Characterization Methods
**Rheological behavior (storage modulus G′, loss modulus G″)**	Determines viscoelasticity, shape retention, and mechanical stability.	Appropriate viscoelasticity improves cell infiltration, proliferation, injectability, and conformal wound coverage while maintaining hydrogel integrity under physiological stress.	Oscillatory rheometry (frequency/amplitude sweep)
Crosslink density	Regulates network architecture, pore size, and mesh structure.	Controls swelling, degradation, drug diffusion, mechanical strength, and cellular migration, thereby influencing tissue regeneration.	Swelling analysis, FTIR, NMR, mechanical testing
Swelling behavior	Determines fluid absorption and moisture retention.	Maintains a moist wound environment, absorbs excess exudate, facilitates nutrient transport, and supports cell migration and proliferation.	Gravimetric swelling ratio
Gel fraction	Indicates crosslinking efficiency and network stability.	Higher gel fraction improves structural integrity, minimizes polymer dissolution, and enables sustained drug release.	Solvent extraction followed by gravimetric analysis
Degradation kinetics	Determines hydrogel residence time and replacement by regenerating tissue.	Controlled degradation synchronizes scaffold resorption with new tissue formation and prolonged therapeutic delivery.	Enzymatic degradation, mass loss studies
Adhesive strength	Measures hydrogel adhesion to wet tissues.	Improves dressing fixation, reduces dressing displacement, minimizes secondary injury during removal, and enhances patient comfort.	Lap shear, tack adhesion, peel strength
Injectability	Enables minimally invasive administration and in situ gelation.	Facilitates complete filling of irregular wounds while protecting encapsulated cells and bioactive molecules during injection.	Syringe extrusion force, injectability testing
Fatigue resistance/self-recovery	Reflects resistance to repeated deformation.	Maintains long-term structural integrity under body movement and repeated mechanical loading, improving durability during wound healing.	Cyclic compression/tensile rheological recovery tests
Sterilization stability	Evaluates resistance to sterilization processes.	Preserves polymer structure, mechanical properties, swelling behavior, and bioactivity after sterilization, ensuring clinical safety.	Gamma irradiation, EtO, steam sterilization, FTIR, DSC
Long-term structural integrity	Determines storage stability and functional lifespan.	Ensures sustained mechanical support, controlled drug release, and consistent wound healing performance throughout treatment.	Accelerated stability testing, mechanical/rheological evaluation

**Table 16:** Clinical trial status of biopolymer-based hydrogels, including study type/phase, study description, enrolment, and clinical trial identifier.

Clinical Trials	Study Type/ Phase	Description	Enrolment	Identifier
Assessment of Wound Healing, Cooling Efficacy and Local Tolerability of a Wound Care Hydrogel	Interventional	The study evaluated the wound healing efficacy, cooling effect, and tolerability of the wound care gel through re-epithelialization assessment, cooling after first application, cosmetic outcomes, participant feedback, and healing time.	33	NCT06309446
Clinical Trial Using the Proteolytic Fraction P1G10 From V. Cundinamarcensis to Heal Diabetic Foot Ulcer (P1G10)	Phase 2	The study evaluated the efficacy and safety of 0.1% P1G10 derived from *Vasconcellea cundinamarcensis* in treating chronic diabetic foot ulcers compared with a standard hydrogel dressing containing water, carboxymethyl cellulose, and sodium alginate.	50	NCT03700580
Comparing How Burn Wounds and Scars Heal in Children Using Chitosan and Silver Dressings (CHITAG)	Interventional	The randomized, open-label, two-arm study compared chitosan-based and silver-based dressings in pediatric burn patients to evaluate burn wound healing, scar formation, cost-effectiveness, and patient experience.	40	NCT06987981
Comparative Efficacy of ADM Hydrogel vs. Alginate Dressings in Chronic Trauma Wounds	Interventional	The clinical trial evaluated the effectiveness of an injectable acellular dermal matrix hydrogel in promoting healing of chronic traumatic wounds in adults compared with standard alginate dressings.	130	NCT06978569
Clinical Trial Evaluating the Safety and Efficacy of the Use of Chitosan Gel in Patients with Chronic Wounds (CHITOWOUND)	Interventional	The multicentre, randomized, double-blind, placebo-controlled trial evaluated the safety and efficacy of chitosan gel for chronic wound treatment.	46	NCT04178525
Treatment of Full-Thickness Wounds: NPWT Combined with Type-I Collagen-Based Advanced Skin Substitute Versus NPWT Alone	Interventional	The study compared the efficacy of NPWT combined with High-Purity Type-I Collagen versus NPWT alone in promoting healing of full-thickness wounds.	104	NCT06873867

## Data Availability

No new data were created or analyzed in this study.

## Abbreviations

The following abbreviations are used in this manuscript:

BC	Bacterial cellulose
CMC	Carboxymethyl cellulose
CMCS	Carboxymethyl chitosan
CMS	Carboxymethyl starch
CNCs	Cellulose nanocrystals
EGF	Epidermal growth factor
E. coli	Escherichia coli
ECM	Extracellular matrix
EVs	Extracellular vesicles
FGF	Fibroblast growth factor
GelMA	Gelatin methacryloyl
GMP	Good manufacturing practice
HA	Hyaluronic acid
HEC	Hydroxyethyl cellulose
HPMC	Hydroxypropyl methylcellulose
IL-1	Interleukin-1
IL-6	Interleukin-6
IL-10	Interleukin-10
MMP	Matrix metalloproteinases
MRSA	Methicillin-resistant S. aureus
NC	Nanocelullose
NF-κB	Nuclear factor- κB
NIR	Near-infrared
OC	Oxidized cellulose
PAAm	Polyacrylamide
P. aeruginosa	Pseudomonas aeruginosa
PDGF	Platelet-derived growth factor
PEG	Polyethylene glycol
PNIPAAm	Poly (N-isopropyl acrylamide)
PU	Poly urethane
PVA	Polyvinyl alcohol
PVP	Poly (vinyl pyrrolidine)
QCS	Quaternized chitosan
ROS	Reactive oxygen species
S. aureus	Staphylococcus aureus
SA	Sodium alginate
SBMDS	Substance-based medical devices
TGF-β	Transforming growth factor-beta
TNF-α	Tumor necrosis factor-alpha
VEGF	Vascular endothelial growth factor
